# Comparative proteomic analysis of wall-forming bodies and oocyst wall reveals the molecular basis underlying oocyst wall formation in *Eimeria necatrix*

**DOI:** 10.1186/s13071-023-06076-6

**Published:** 2023-12-18

**Authors:** Lele Wang, Dandan Liu, Yu Zhu, Feiyan Wang, Weimin Cai, Qianqian Feng, Shijie Su, Zhaofeng Hou, Jinjun Xu, Junjie Hu, Jianping Tao

**Affiliations:** 1 College of Veterinary Medicine, Yangzhou University, 12 East Wenhui Road, Yangzhou, 225009 Jiangsu People’s Republic of China; 2 Jiangsu Co-Innovation Center for Prevention and Control of Important Animal Infectious Diseases and Zoonoses, Yangzhou University, Yangzhou, 225009 People’s Republic of China; 3 Joint International Research Laboratory of Agriculture and Agri-Product Safety, The Ministry of Education of China, Yangzhou University, Yangzhou, 225009 People’s Republic of China; 4 School of Ecology and Environmental Sciences and Yunnan Key Laboratory for Plateau Mountain Ecology and Restoration of Degraded Environments, Yunnan University, Kunming, 650091 People’s Republic of China

**Keywords:** *Eimeria necatrix*, Wall-forming bodies, Oocyst wall, Tandem mass tag, Comparative proteomics, Differentially expressed proteins

## Abstract

**Background:**

The durable oocyst wall formed from the contents of wall-forming bodies (WFBs) protects *Eimeria* parasites from harsh conditions and enhances parasite transmission. Comprehending the contents of WFBs and proteins involved in oocyst wall formation is pivotal to understanding the mechanism of the oocyst wall formation and the search for novel targets to disrupt parasite transmission.

**Methods:**

Total proteins extracted from WFBs and the oocyst wall of *Eimeria necatrix* were subjected to comparative proteomic analysis using tandem mass tag in conjunction with liquid chromatography tandem-mass spectrometry techniques. After functional clustering analysis of the identified proteins, three proteins, including *E. necatrix* disulfide isomerase (EnPDI), thioredoxin (EnTrx) and phosphoglycerate kinase (EnPGK), were selected for further study to confirm their potential roles in oocyst wall formation.

**Results:**

A total of 3009 and 2973 proteins were identified from WFBs and the oocyst wall of *E. necatrix*, respectively. Among these proteins, 1102 were identified as differentially expressed proteins, of which 506 were upregulated and 596 downregulated in the oocyst wall compared to the WFBs. A total of 108 proteins, including compositional proteins of the oocyst wall, proteases, oxidoreductases, proteins involved in glycosylation, proteins involved in synthesis of the acid-fast lipid layer and proteins related to transport, were proposed to be involved in oocyst wall formation. The approximate molecular sizes of native EnPDI, EnTrx and EnPGK proteins were 55, 50 and 45 kDa, respectively. EnPDI was present in both type 1 and type 2 WFBs, EnTrx was present only in type 2 WFB2 and EnPGK was present only in type 1 WFBs, whereas all of them were localized to the outer layer of the oocyst wall, indicating that all of them participate in the formation of the oocyst wall.

**Conclusions:**

To the best of our knowledge, this is the first report on the proteomes of WFBs and the oocyst wall of *E. necatrix*. The data obtained from this study form a basis for deciphering the molecular mechanisms underlying oocyst wall formation of *Eimeria* parasites. They also provide valuable resources for future studies on the development of novel therapeutic agents and vaccines aimed at combating coccidian transmission.

**Graphical Abstract:**

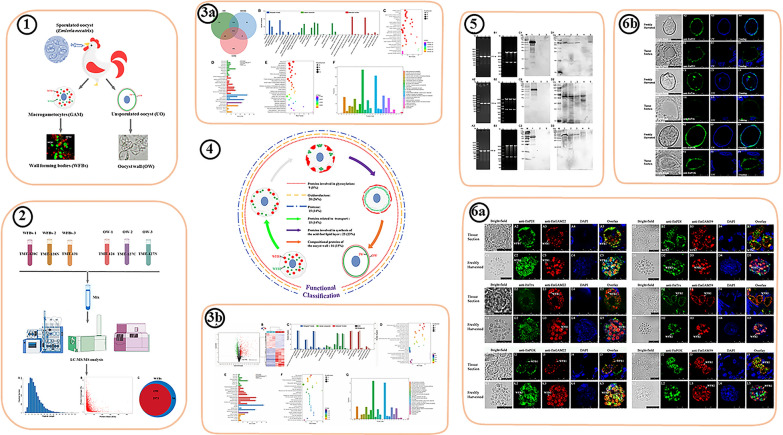

**Supplementary Information:**

The online version contains supplementary material available at 10.1186/s13071-023-06076-6.

## Background

Avian coccidiosis, a parasitic disease caused by one or more of seven species of the genus *Eimeria*, is one of the most widely reported diseases within the poultry industry worldwide [1, 2]. *Eimeria necatrix* is highly pathogenic and causes major lesions in the small intestine and substantial mortality, particularly in chickens older than 8 weeks raised on litter floors [1]. Conventional coccidiosis control strategies rely heavily on chemoprophylaxis and, to a certain extent, live vaccines [3]. Annually, the poultry industry spends about £7.7 to £13.0 billion (at 2016 prices) in only seven countries on prophylaxis, treatment and production losses due to avian coccidiosis [4]. In this context, avian coccidiosis remains one of the major problems globally to date.

*Eimeria* parasites undergo complex life-cycles involving asexual proliferation followed by sexual development leading to the production of oocysts [1]. Prior to excretion in the feces of the infected hose, the oocyst is encapsulated by a hard barrier, the oocyst wall, which protects the parasite from the harsh external environment. Once excreted from the host, the oocyst develops further (sporulation) and is passed onto the next host via the fecal–oral route [5]. The oocyst wall is vital for parasite survival in the external environment [5, 6]. Therefore, a good understanding of the molecular basis of oocyst wall formation may be relevant to the development of novel vaccines and drugs for treating the debilitating diseases caused by *Eimeria* parasites, as well as those caused by the related cyst-forming parasites *Toxoplasma gondii*, *Cryptosporidium* spp. and *Neospora caninum*, among others.

A great deal of research has focused on elucidating the structure, biochemical composition and developmental biology of the oocyst wall of *Eimeria* spp. [5–7]. This research has so far revealed that the bilayered oocyst wall is formed from the contents of two organelles, wall-forming bodies type 1 and 2 (WFB1, WFB2), located exclusively in the macrogametocytes [8]. WFB1s and WFB2s are synthesized during the maturation of the macrogametes and appear to give rise to the outer and inner layers of the oocyst wall, respectively [6]. Compositional analysis of *E. tenella* and *E. maxima* showed that their oocyst walls were primarily composed of protein (> 90% of wall) and only relatively small amounts of lipid and carbohydrate [5, 6]. However, only a small number of oocyst wall proteins have been identified and characterized to date, and these are mainly tyrosine-rich proteins ranging in size from 8 to 31 kDa [9]. Studies have revealed that these tyrosine-rich proteins are all derived from precursor proteins stockpiled in WFBs of macrogametes. During oocyst wall formation, these precursor proteins are proteolytically processed into smaller tyrosine-rich proteins prior to protein–tyrosine cross-linking and hardening of the oocyst wall [5]. However, the contents of WFBs and the identity of the proteins and enzymes involved in oocyst wall formation remain poorly understood.

In the present study, we employed tandem mass tag (TMT) peptide labeling coupled with the liquid chromatography tandem mass spectrometry (LC–MS/MS) quantitative proteomics technique to investigate the protein abundance of WFBs and the oocyst wall of *E. necatrix*. Previous studies had revealed that protein disulfide isomerase (PDI) and thioredoxin (Trx) are involved in disulfide oxidoreduction and contribute significantly to cyst wall formation in *Giardia* [10]. Trxs and PDI, both members of the thioredoxin superfamily, share a common thioredoxin fold and play roles in disulfide oxidoreduction and/or isomerization [11]. Additionally, phosphoglycerate kinase (PGK) is a bona fide cell wall protein of *Candida albicans* [12] and is crucial for cytoskeletal rearrangements in *Schistosoma mansoni* [13]. In this context, we aimed to determine whether *E. necatrix* PDI (EnPDI), EnTrx and EnPGK are involved in the oocyst wall formation of *E. necatrix*. We also aimed to identify the proteins potentially involved in oocyst wall formation. The results of this study would provide important information on oocyst wall formation and also contribute to new targets for avian coccidiosis control.

## Methods

### Parasites and animals

The *E. necatrix* Yangzhou strain used in this study was originally isolated from a chicken that died of coccidiosis caused by *E. necatrix* in 2009 in Yangzhou, China, as confirmed by microscopic examination and sequence analysis of the internal transcribed spacer region of extracted genomic DNA. This strain has been maintained in our laboratory according to the method previously described [14].

One-day-old chickens were obtained from the Poultry Institute of the Chinese Academy of Agricultural Sciences (Yangzhou, Jiangsu, China). The chickens were housed in *Eimeria*-free isolation cages and provided with clean water and adequate feed without anticoccidial drugs. Chicken feces were collected and analyzed by salt flotation and light microscopy to confirm the absence of oocysts in the chickens 1 day before the experimental inoculations. Chickens between 4 and 5 weeks of age were used to prepare gametocytes (GAM) and oocysts of *E. necatrix*. Six-week-old specific-pathogen-free female BALB/c mice were purchased from Yangzhou University (Comparative Medicine Center) and maintained under specific-pathogen-free conditions. These mice were used to prepare the antibodies against recombinant proteins.

All animal care and procedures were conducted according to the guidelines for animal use in toxicology. The study protocol was approved by the Animal Care and Use Committee of the College of Veterinary Medicine, Yangzhou University.

### GAM preparation and WFB isolation

Gametocytes and WFBs were obtained following the method described previously [15]. Briefly, *E. necatrix* second-generation merozoites (MZ-2) were obtained from the small intestine of chickens 136 h after oral inoculation with 2.0 × 10^4^ *E. necatrix* sporulated oocysts. Following the administration of halothane to induce anesthesia, the ceca of chickens were exposed and the base of the ceca tied off with a cotton ligature. Subsequently, approximately 1.8 × 10^8^ MZ-2 in a volume of 1.5–2 ml were injected into the cecal lumen using a syringe with a 25-gauge needle. At 30 ± 0.5 h after injection with MZ-2, the chickens were sacrificed and the ceca removed. The mucosal tissues were obtained and digested with hyaluronidase in SAC (1 mM phenylmethanesulfonyl fluoride, 1 mg/ml bovine serum albumin [BSA], 170 mM NaCl, 10 mM Tris–HCl pH 7.0, 10 mM glucose and 5 mM CaCl_2_) to isolate the GAM. The GAM were purified using Percoll (GE Healthcare, Uppsala, Sweden) density gradient centrifugation as follows. First, 2 ml of 50% Percoll/PBS (volume ratio 1:1) solution was placed into a sterile 15-ml tube, followed by the slow addition of 5 ml of 30% Percoll/GAM suspension (volume ratio 3:7). The tube was then centrifuged at 3000 *g* for 20 min, following which the supernatant was discarded, and the GAM washed twice with cold PBS by centrifugation. The purified GAM (1 × 10^8^ cells) were then extracted with 0.1% saponin in Tris-NaCl-EDTA (TNE) buffer for 20 min at room temperature and centrifuged at 1000 *g* for 5 min. After washing with TNE buffer, the pellet was sonicated in an ice water bath and the lysates filtered through a 5-μm polymon mesh. The filtrate was then added to 5% sodium dodecyl sulfate, vortexed and centrifuged at 15,000 *g* for 10 min. The WFBs in the pellet were purified using a 1000-kDa cutoff Vivaspin 6 centrifugal filter (Sartorius Stedim Biotech, Aubagne, France), centrifuged at 4000 *g* for 20 min and concentrated three times. The purified WFBs were obtained from the concentrated solution by centrifugation at 15,000 *g* at 4 °C for 10 min. Finally, WFB1 and WFB2 were confirmed by immunofluorescence co-localization using anti-recombinant* E. necatrix* GAM protein 22 (anti-rEnGAM22) and anti-recombinant* E. necatrix* GAM protein 59 (anti-rEnGAM59) polyclonal antibody (pAb) [15]. The purified WFBs were stored at 4 °C or frozen immediately in liquid nitrogen for future use. Three biological replicates were performed to collect WFBs for proteomic analysis.

### Isolation and purification of the oocyst wall

Oocysts were isolated and purified according to the method reported previously [14]. Briefly, the 4– to 5-week-old chickens were orally infected with approximately 2 × 10^4^ sporulated oocysts of *E. necatrix*. The feces were collected between 6 and 9 days after infection to separate oocysts out using a saturated salt solution and saturated sucrose solution, respectively. After the purified oocytes had been sterilized by sodium hypochlorite treatment and washed with sterile water, they were used to prepare the oocyst wall according to the previously described method [16] with modifications. Briefly, approximately 4 × 10^7^ purified unsporulated oocysts were disrupted by vigorous vortexing in 2 ml of PBS containing 1 mM PMSF, 1 mM EDTA and 1 mM DTT and 2 g of acid-washed glass beads (≤ 106 μm; Sigma-Aldrich, St. Louis, MO, USA) for 5 min until > 80% of them were ruptured. The resulting mixture was resuspended in 5 ml of 0.5 M sucrose and layered onto 3 ml of 1.1 M sucrose in a 15-ml centrifuge tube and centrifuged at 3000 *g* for 15 min. The pellet was washed 5 more times by resuspension in 10 vol of PBS and centrifugation at 10,000 *g*. The final pellet, consisting of purified oocyst wall, was then lash-frozen in liquid nitrogen. Three biological replicates were performed to collect the oocyst wall for proteomic analysis.

### TMT-based quantitative proteomics analysis

#### Pre-treatment and TMT labeling of protein

Samples of WFBs and the oocyst wall were resuspended in lysis buffer containing 8.0 M urea, 0.1% SDS, 1× Protease Inhibitor Cocktail tablets (Roche, Indianapolis, IN, USA) and 1 mM PMSF (Biyotime Institute of Biotechnology, Shanghai, China) [17, 18]. After incubation on ice for 30 min, the lysed samples were centrifuged, followed by concentration through a 3-kDa filter (Amicon Ultra-0.5 Centrifugal Filter Unit with Ultracel-3 membrane; MilliporeSigma, Burlington, MA, USA), and the resulting supernatant was stored at − 80 °C [17, 19]. Protein concentration was determined using the Pierce BCA Kit (Thermo Fisher Scientific, Waltham, MA, USA) according to the manufacturer’s protocol. Efficient protein extraction was confirmed via electrophoresis, with equal amounts (20 μg) of protein loaded onto an SDS-polyacrylamide gel electrophoresis (PAGE) gel and subsequent staining of the products with Coomassie Brilliant Blue R-250.

To generate peptides, the protein extracted from each sample was digested with 2.5 μg of trypsin (Thermo Fisher Scientific). These resulting peptides were then labeled using the TMT kit (TMT 10 plex™ Isobaric Label Reagent Set; Thermo Fisher Scientific). Specifically, peptides within each experimental group were labeled with distinct TMT labels: three biological replicates of the oocyst wall group were designated as TMT-126, TMT-127C and TMT-127N, while three biological replicates of the WFB group were labeled as TMT-128C, TMT-128N and TMT-131.

#### High-performance liquid chromatography fractionation and LC–MS/MS analysis

The TMT-labeled peptides were dissolved in solvent A buffer (98% double-distilled water, 2% acetonitrile, pH 10) and fractionated by high-pH reversed-phase fractionation chromatography utilizing an XBridge C18 column (5 μm, 250 × 4.6 mm; Waters Corporation, Milford, MA, USA). Fractions were collected every 1 min in 40 tubes, subsequently dehydrated and then amalgamated into 10 tubes to facilitate subsequent LC–MS/MS analysis.

LC–MS/MS analysis was performed on the U3000 Nano-Scale Liquid Chromatography System (Thermo Fisher Scientific) coupled with a Q-Exactive mass spectrometer (Thermo Fisher Scientific) via a nano-electrospray source. TMT-labeled peptides were loaded onto a 25-cm-long, 75-μm inner diameter fused silica analytical column packed with 2.0 μm Aqua C18beads (Thermo Fisher Scientific), using an autosampler at 5 μl/min. Elution utilized a gradient of buffer A (0.1% formic acid in water) and buffer B (0.1% formic acid in acetonitrile) at a flow rate of 300 nl/min for 43.5 min. Ion signals were acquired in data-dependent mode, with a full scan resolution of 70,000 and a scan range of* m/z* 350–1600. The resulting MS/MS data were saved as raw files using Xcalibur software version 2.2 (Thermo Fisher Scientific). TMT-proteomic analysis was conducted by CapitalBio Corporation, Beijing, China. The proteomics data generated by mass spectrometry have been submitted to the ProteomeXchange Consortium through the PRIDE partner repository under the dataset identifier PXD042839 and can be accessed at https://www.ebi.ac.uk/pride/archive.

#### Protein identification and quantitative analysis

The raw MS data were analyzed using Proteome Discoverer 2.3 software, which was used for database retrieval, peptide mapping and protein quantitation. The MS/MS spectra were searched against five reference databases, namely those of *E. necatrix* (https://www.ncbi.nlm.nih.gov/assembly/GCF_000499385.1/), *E. tenella* (https://www.ncbi.nlm.nih.gov/assembly/GCF_000499545.2/), *E. maxima* (https://www.ncbi.nlm.nih.gov/assembly/GCF_000499605.1/), *T. gondii* (https://www.ncbi.nlm.nih.gov/assembly/GCF_000006565.2/) and *Plasmodium falciparum* (https://www.ncbi.nlm.nih.gov/assembly/GCF_000002765.4/). To control the false discovery rate (FDR) at the protein and peptide levels, we applied a fusion-decoy database search strategy with a threshold of ≤ 1.0%. Confident protein identifications required a minimum of two unique peptides with at least two corresponding spectra. Protein expression levels were quantified for each sample, utilizing the combined intensities of the three most prominent ion peaks from tryptic peptides, which enabled effective comparisons. Proteins and peptide features with a fold change ≥ 1.5 and a *P*-value < 0.05 between the WFB and oocyst wall groups were designated as differentially expressed proteins (DEPs) for further bioinformatics analysis.

#### Bioinformatic analysis of proteins

Gene Ontology (GO) (http://www.geneontology.org/; accessed date: March 2019) and Kyoto Encyclopedia of Genes and Genomes (KEGG) pathway analyses (http://www.genome.jp/kegg/pathway.html; accessed date: March 2019) were performed for functional annotation and pathway assessment of the total identified proteins and DEPs using KOBAS 2.0 software (http://kobas.cbi.pku.edu.cn/kobas3; accessed date: March 2019) [20–23]. The *P*-value was set as ≤ 0.05 as the threshold to judge the significance of the GO and KEGG pathway enrichment analyses. Furthermore, the Cluster of Orthologous Groups of proteins (COG; https://www.ncbi.nlm.nih.gov/COG/ accessed date: March 2019) database was used by Blastall software (version 2.2.25) to classify and group the proteins [24].

#### Validation of proteomics data

To ensure the credibility of the TMT data, we performed Simple Western analysis using the WES™ automated instrument (ProteinSimple, San Jose, CA, USA) in accordance with the manufacturer’s protocols, to analyze the EnGAM22 and EnGAM59 protein expression profiles in the oocyst wall and WFBs. The proteins extracted from WFBs and the oocyst wall were diluted to a final concentration of 0.5 μg/μl with 5× Master Mix (ProteinSimple). Primary rabbit pAb against EnGAM22 and EnGAM59, prepared as described previously [15], were diluted 1:50 in Antibody Diluent II (ProteinSimple), and secondary antibodies were applied according to the manufacturer’s instructions. Following automated separation and immunodetection, Compass software (ProteinSimple) was employed to visualize and analyze signal peaks, generating relative protein quantification from sample chromatograms and virtual gel images.

### Identification of proteins potentially involved in the oocyst wall formation

#### Cloning and expression of EnPDI, EnTrx and EnPGK

RNA was extracted from purified gametocytes using the FastPure Cell/Tissue Total RNA Isolation Kit V2 (Vazyme, Nanjing, China), followed by reverse transcription using the HiScript III First Strand cDNA Synthesis Kit (+ gDNA wiper) (Vazyme). Three genes, including En*PDI* (protein disulfide isomerase; GenBank accession number: ENH_00036560), En*Trx* (thioredoxin; GenBank accession number: ENH_00002620) and En*PGK* (phosphoglycerate kinase; GenBank accession number: ENH_00071160), were amplified by PCR with Premix TaqTM (TakaRa, Tokyo, Japan) and three pairs of specific primers (Table 1), respectively. The PCR products were cloned into the pGEM-T-easy vector (Promega Corp., Madison, WI, USA), and the protein sequences were predicted and analyzed using Lasergene 7.0 and Clustal X.

**Table 1:** Primer sequence

Primer name	Primer sequence 5ʹto 3ʹ^c^
En*PDI*-F^a^	ATGAAAAGACCTTTCTTGCTCGG
En*PDI*-R^a^	TCAGAGTTCCTCGCCCTTGTC
En*PDI*-pET28aF^b^	*CG*GGATCCGCAGCAGCAGCAGCAGAGAACAA
En*PDI*-pET28aR^b^	*CCG*CTCGAGTCAGAGTTCCTCGCCCTTGTCGG
En*Trx*-F^a^	ATGGCTTTGGGCGTTGGCTTAGGAA
En*Trx*-R^a^	CTAAAGCTCTTCTTTCTTTGTGT
En*Trx*-pET28aF^b^	*CG*GGATCCGCGCGGCCGCTGATCGACCCCAT
En*Trx*-pET28aR^b^	*CG*GAATTCCTAAAGCTCTTCTTTCTTTGTGT
En*PGK*-F^a^	ATGCGCGTGGACTTCAACGTGCC
En*PGK*-R^a^	TCACTTTGAAGACAAAGCTGCCACTC
En*PGK*-pET28aF^b^	*CG*GGATCCATGCGCGTGGACTTCAACGTGCC
En*PGK*-pET28aR^b^	*ATAGTTTA*GCGGCCGCTCACTTTGAAGACAAAGCTG

*En Eimeria necatrix*,* F* forward primer,* PDI* protein disulfide isomerase,* pET28a* bacterial vector,* PGK* phosphoglycerate kinase,* R* reverse primer,* Trx* thioredoxin

^a^Primers for complete gene

^b^Primers for prokaryotic expression

^c^The protective bases are shown in italics and the restriction sites are underlined

The En*PDI*, En*Trx* and En*PGK* genes, excluding the signal peptide sequence, were then amplified using three pairs of specific primers with restriction enzyme sites *Bam*HI and *Xho*I, *Bam*HI and *Eco*RI and *Bam*HI and *Not*I, respectively (Table 1). The PCR products of En*PDI*, En*Trx* and En*PGK* were subcloned into the pET28a(+) bacterial expression vector (Invitrogen, Thermo Fisher Scientific), then transformed into BL21 (DE3) cells (TransGen Biotech Co., Ltd, Beijing, China) and induced to express the recombinant proteins by adding 1 mM isopropyl β-d-1-thiogalactopyranoside (IPTG) and incubation for 12 h at 37 °C. The recombinant proteins EnPDI, EnTrx and EnPGK (rEnPDI, rEnTrx and rEnPGK) were subsequently purified using High-Affinity Ni–NTA Resin (GenScript, Piscataway, NJ, USA) according to the manufacturer’s instructions. Purified recombinant proteins were then incubated with anti-6×His tag mouse monoclonal antibody (dilution: 1:500; Beyotime, Shanghai, China) for detection.

#### Detection of native protein EnPDI, EnTrx and EnPGK

The proteins of MZ-2, third-generation merozoites (MZ-3) (purified as previously reported [25]), GAM and unsporulated and sporulated oocysts (UO and SO) were abstracted using the method described previously [15], and their concentrations were determined as described above. Subsequently, 10 μg of total protein lysates was separated by 12% SDS-PAGE and transferred to nitrocellulose membranes (MilliporeSigma). The membranes were blocked with 3% BSA (Merck & Co., Rahway, NJ, USA) in PBS overnight at 4 °C, then incubated respectively with anti-rEnPDI, rEnTrx or rEnPGK mouse pAb (1:400 dilution) prepared by the method described previously [15]. Following the incubation, the membranes were washed 3 times with 0.03% Tween-20/TBS (TBST) for 10 min each time and then probed with peroxidase-conjugated AffiniPure goat anti-mouse immunoglobulin G (IgG) (H+L; 1:10,000; Jackson ImmunoResearch Labs, West Grove, PA, USA) for 45 min at 37 °C. Finally, the membranes were developed in the presence of High-sig ECL Western blotting substrate (Tanon, Shanghai, China) after washing with TBST. Naïve sera from mice were used as a negative control.

#### Localization of EnPDI, EnTrx and EnPGK in GAM and UO

Indirect immunofluorescence analyses (IFA) were performed on tissue samples and purified parasites to investigate the localization of EnPDI, EnTrx and EnPGK proteins in *E. necatrix* according to the method reported in our previous study [15]. Briefly, pathological tissue samples obtained from chickens sacrificed 156 h post-infection and from GAM and UO were immobilized on poly-L-lysine-coated glass slides and treated with 1.0% Triton X-100 for 20 min at room temperature. To quench endogenous peroxidase activity in the pathological tissue samples, the samples were incubated with 3% H_2_O_2_ for 30 min, and antigen retrieval was accomplished by a 10-min exposure to 1 mg/ml trypsin (diluted with 0.1 M CaCl_2_). Following fixation of both tissue samples and purified parasites in methanol (− 20 °C) and subsequent blocking with 5% BSA, all samples were respectively incubated with anti-rEnPDI, rEnTrx or rEnPGK mouse pAb (1:100 dilution) at 37 °C for 1 h. Additionally, anti-rEnGAM22 rabbit pAb (1:100 dilution) and anti-rEnGAM59 rabbit pAb (1:100 dilution) were used as markers for WFB1 and WFB2 in the co-localization studies [15]. After washing with 0.03% Tween 20/PBS (PBST), the samples were incubated with FITC-conjugated goat anti-rabbit IgG (1:100 dilution; MultiSciences, Hangzhou, China) and Cy3-conjugated goat anti-mouse IgG (1:100 dilution; Servicebio, Wuhan, China) in BSA/PBS at 37 °C for 1 h. Subsequently, the samples were visualized by laser (point) scanning confocal microscopy (LCM; Leica TCS SP8 STED microscope; Leica Microsystems GmbH, Wetzlar, Germany). Naïve sera from rabbits and mice served as negative controls.

## Results

### Observation and SDS-PAGE analysis of the oocyst wall and WFBs

**Fig. 1:**
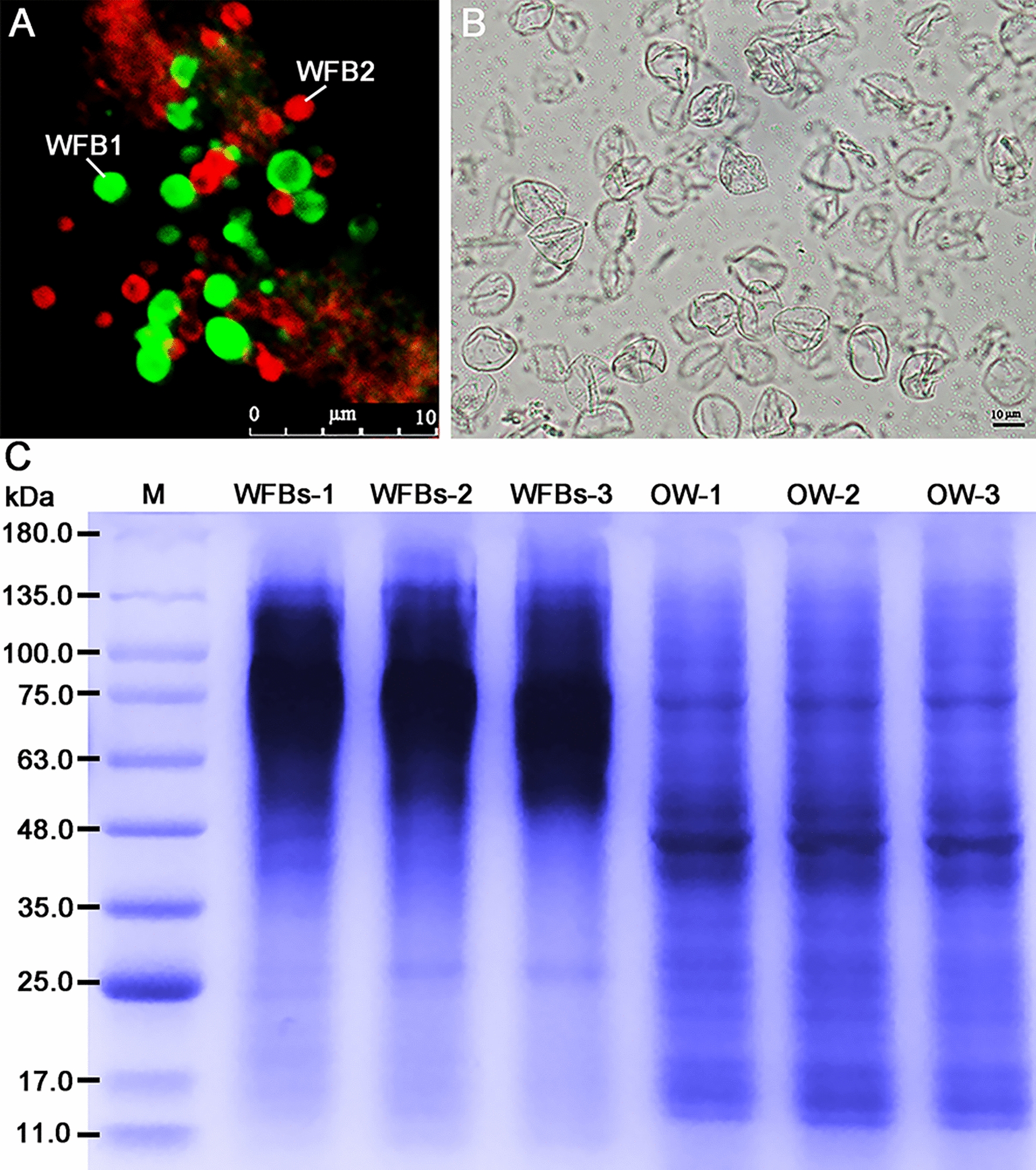
Microscopy observation and SDS-PAGE analysis of the purified WFBs and the oocyst wall. **a** Immunofluorescence co-staining of the WFB-rich extract incubated with rabbit anti-rEnGAM22 pAb (visualized with FITC, green) and mouse anti-rEnGAM59 pAb (visualized with Cy3, red), showing that the enriched fractions include both intact WFB1s and intact WFB2s. **b** Purified oocyst wall of *Eimeria necatrix* observed by light microscope. **c** SDS-PAGE analysis of three biological repetitions of WFBs and OWs. Lanes: M, Marker; WFBs-1/WFBs-2/WFBs-3, WFB replicates 1/2/3; OW-1/OW-2/OW-3, OW replicates 1/2/3. Scale bars: 10 μm. Anti-rEnGAM22/-rENGAM59, Anti-recombinant* E. necatrix* GAM protein 22/GAM protein 59; GAM, Gametocyte(s); OW, oocyst wall; pAb, polyclonal antibody; SDS-PAGE, sodium dodecyl sulfate-polyacrylamide gel electrophoresis; WFB1/2, wall-forming body type 1/type 2

In our previous study, we demonstrated that anti-rEnGAM22 antibody specifically binds to WFB1s, while the anti-rEnGAM59 antibody specifically binds to WFB2s [15]. In the present study, our double-immunofluorescence labeling analysis confirmed the presence of two types of WFBs in our isolations (Fig. 1a). Light microscopy revealed that the oocyst walls preparations had minimal contamination (Fig. 1b), and the BCA assay showed that the protein concentrations were similar in the three replicates of the oocyst wall preparations (5.316, 4.670 and 4.935 μg/μl, respectively) and the WFB preparations (3.096, 2.967 and 3.057 μg/μl, respectively). SDS-PAGE analysis showed that the protein bands mainly ranged from 11 to 135 kDa in the oocyst wall extracts and primarily from 35 to 135 kDa in the WFB extracts (Fig. 1c). These results confirmed that the samples were successfully prepared and could be used for further proteomics research.

### Overview of primary data and protein identification

**Fig. 2:**
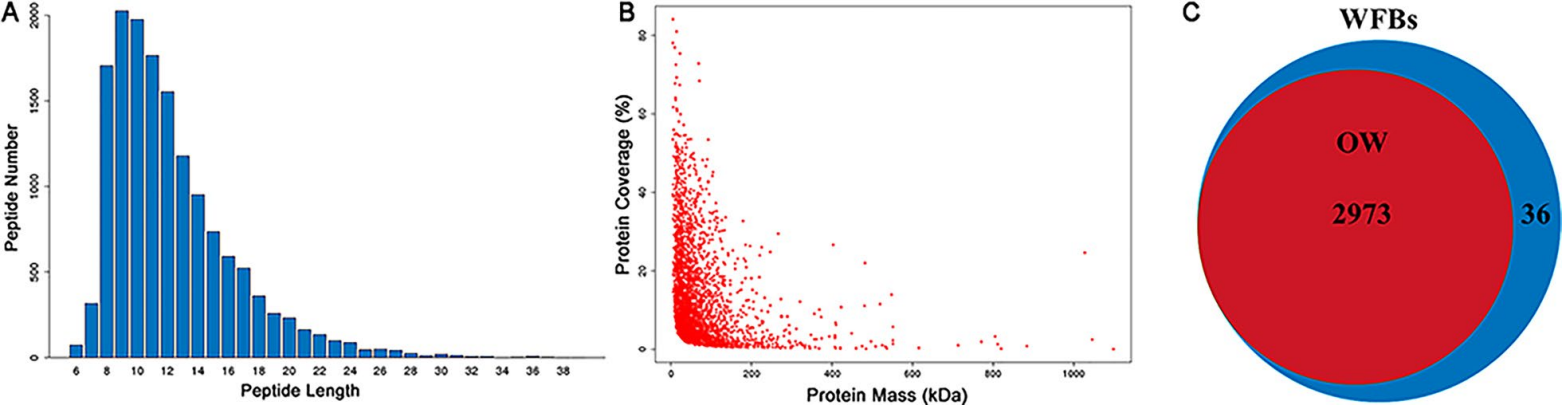
Overview of tandem mass tag-based quantitative proteomics for primary data and protein identification. **a** Distribution of peptide fragment lengths derived from WFBs and OW. **b** Distribution of identified proteins with different molecular weights. **c** Venn diagram showing overlapping proteins between WFBs and OW. OW, Oocyst wall; WFBs, wall-forming bodies

Of the 255,975 detected spectra, 232,083 were successfully identified, resulting in 28,449 peptide spectrum matches (PSMs) that encompassed 12,730 unique peptides. Editing and normalizing the data yielded a total of 3391 proteins having a confident prediction (FDR < 1%) across three biological replicates, of which 3059 proteins could be quantified using ≥ 2 peptides. Peptide length and count distribution revealed a predominant range of 8–13 amino acids (Fig. 2a), and > 80% of the identified proteins had a molecular size of < 200 kDa (Fig. 2b). As shown in Fig. 2c, a total of 2973 proteins were identified in the oocyst wall and 3009 proteins were identified in the WFBs, of which 36 were WFB-specific proteins (Additional file 1: Table S1).

### Protein quantification and differential analysis

**Fig. 3:**
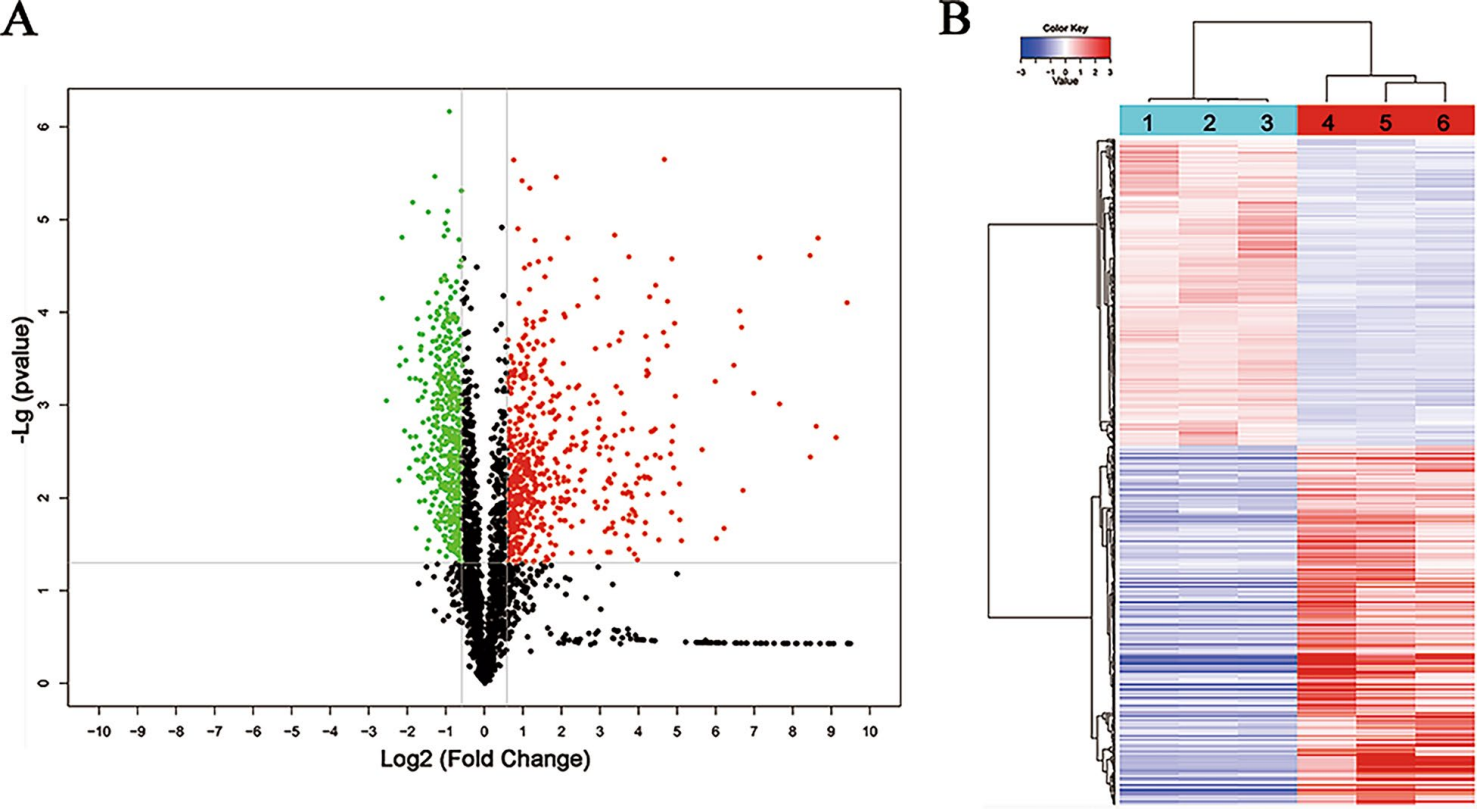
Overview of tandem mass tag-based quantitative proteomics for quantification. **a** Volcano plot of quantified proteins in the proteome database for WFBs and OW. The plot depicts log2 (fold change) on the horizontal axis, with a dashed line indicating the fold-change cutoff. The vertical axis represents log10 (*P*-value), and the dashed line represents the* P*-value cutoff. Red dots represent upregulated proteins in the OW group (fold change ≥ 1.5, *P* < 0.05); green dots represent downregulated proteins in the oocyst wall group (fold change ≤ 0.667, *P* < 0.05); black dots represent proteins with no significant difference in expression (0.667 < fold change < 1.5 or *P* > 0.05). **B** Clustering analysis of the differentially expressed proteins in WFBs and oocyst wall. Lanes: 1, 2, 3, the three biological replicates of the OW; 4, 5, 6, the three biological replicates of WFBs. OW, Oocyst wall; WFBs, wall-forming bodies

Applying a stringent cutoff (1.5-fold change), we identified a total of 1102 DEPs (*P* < 0.05) between WFBs and the oocyst wall (Fig. 3a; Additional file 2: Table S2). These DEPs included 506 upregulated and 596 downregulated proteins in the oocyst wall compared to the WFBs. The hierarchical clustering analysis of DEPs revealed highly similar and closely related expression patterns among the three biological replicates, effectively illustrating the distribution of DEPs and distinguishing the protein expression profiles between WFBs and oocyst wall (Fig. 3b).

### Reliability analysis of proteomics data

**Fig. 4:**
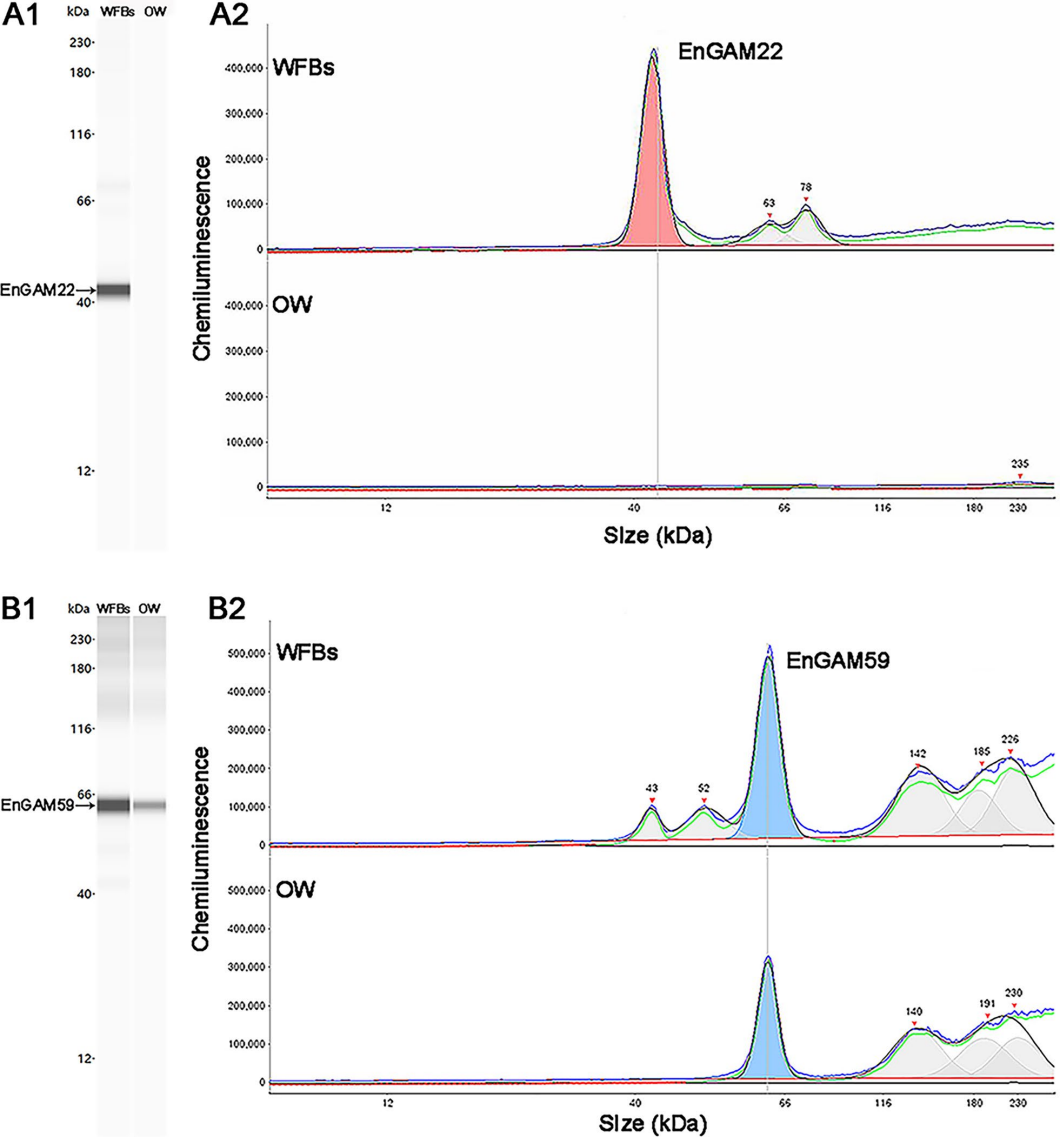
Validation analysis of EnGAM22 and EnGAM59 by immunoblotting (Simple Western analysis).** A1**,** B1** Protein expression levels of EnGAM22 and EnGAM59 in the WFBs and OW groups were analyzed using the capillary-based Simple Western automated system (ProteinSimple) and presented as virtual blots.** A2**,** B2** Relative peak areas of EnGAM22 and EnGAM59 in the WFBs and OW groups were determined using the capillary-based Simple Western automated system. EnGAM22/EnGAM59,* E. necatrix* GAM protein 22/GAM protein 59; OW, oocyst wall; WFBs, wall-forming bodies

The EnGAM22 (AHB64327.1) and EnGAM59 (AKN58547.1) proteins were selected for the validation of protein expression patterns identified by TMT-based quantitative proteomics. The proteomics results revealed downregulation of EnGAM22 in the oocyst wall, whereas there was no significant difference in EnGAM59 expression between the oocyst wall and WFBs. These expression patterns of EnGAM22 and EnGAM59 were confirmed by Simple Western analysis (Fig. 4; Additional file 3: Table S3), which suggested that our proteomic data was reliable.

### Bioinformatic analysis of WFBs protein

**Fig. 5:**
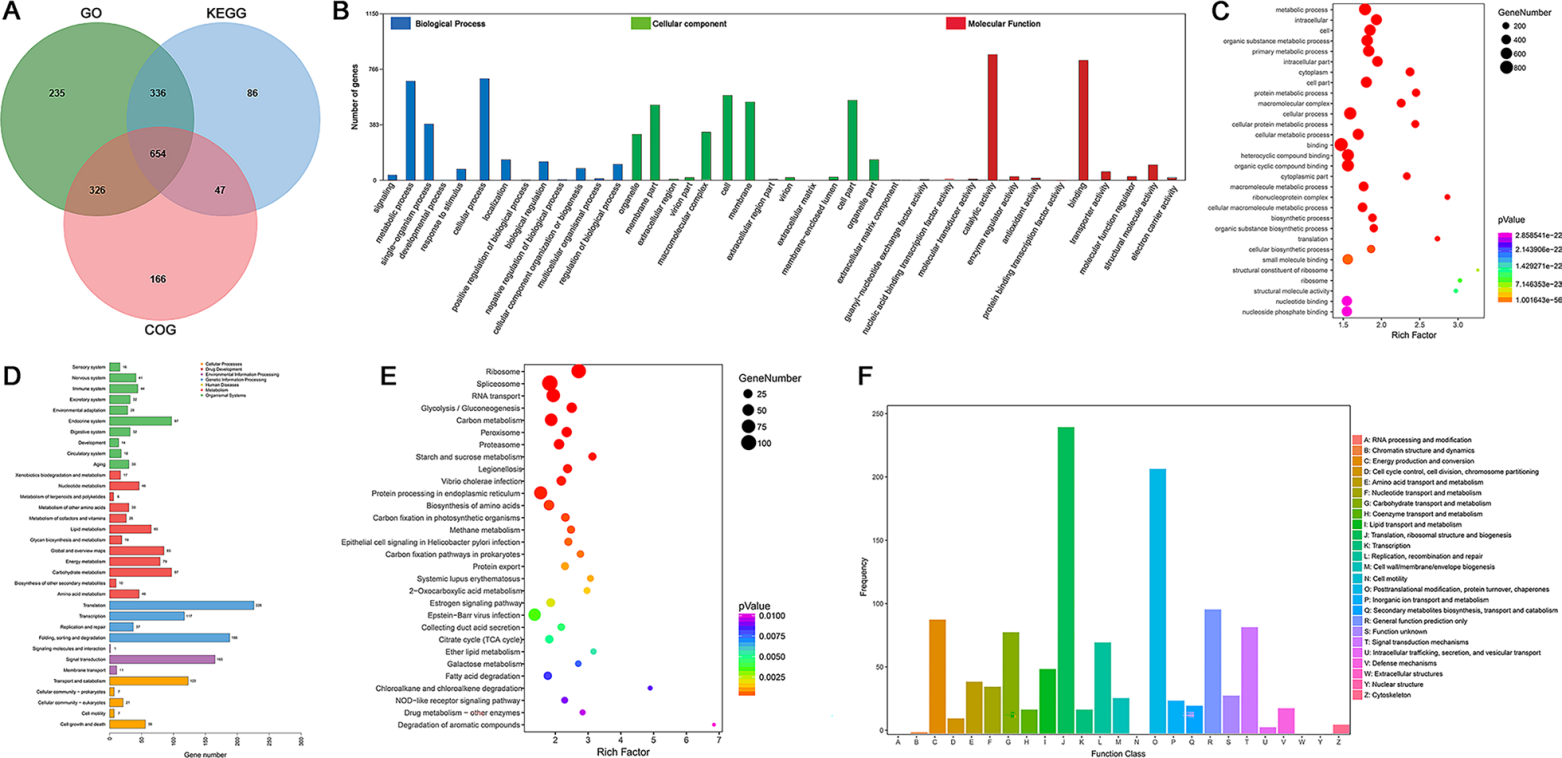
Bioinformatic analysis results of WFB proteins. **a** WFB protein functional annotation results from GO, KEGG and COG databases. Each circle in the diagram represents the annotation result of a database, the overlapping portion represents the protein annotated jointly by multiple databases and the nonoverlapping portion represents the protein annotated separately by the corresponding database. **b** Summary of second-level GO assignments for WFB proteins. The* x*-axis represents the second-level functional categories from the GO database, and the* y*-axis represents the number of WFBs protein in each second-level functional category. **c** Top 30 GO enrichment results for WFB proteins. The* x*-axis represents the results of Input frequency/Background frequency in the enrichment analysis, and the* y*-axis represents enriched GO terms. **d** Summary of second-level KEGG pathway analysis for WFB proteins. The* x*-axis represents the number of genes in each pathway, and the* y*-axis indicates the main second-level pathways. **e** Top 30 KEGG enrichment results for WFB proteins. The* x*-axis represents the results of Input frequency/Background frequency in the enrichment analysis, and the* y*-axis represents enriched KEGG pathway terms. **f** COG function classification of WFB proteins. The* x*-axis indicates the different categories of COG, and the* y*-axis indicates the frequency of different categories of COG. COG, Cluster of Orthologous Groups database; GO, Gene Ontology resource; KEGG, Kyoto Encyclopedia of Genes and Genomes database; WFBs, wall-forming bodies

The proteins identified in both WFBs and the oocyst wall were annotated using the GO, KEGG and COG databases. Of these proteins, 1551 (45.74%), 1123 (33.12%) and 1193 (35.18%) were successfully annotated by GO, KEGG and COG respectively, and 654 of 3391 (19.29%) of proteins were concurrently annotated by all three databases (Fig. 5a). Among the 36 WFB-specific proteins, 15 (41.67%), seven (19.44%) and nine (25%) were successfully annotated by GO, KEGG, and COG, respectively (Additional file 4: Table S4).

The GO analysis of all the WFB proteins assigned 927, 1022 and 1370 proteins to the categories of biological processes (13 GO terms), cellular components (14 GO terms) and molecular function (12 GO terms), respectively (Fig. 5b). The top three GO terms were cellular process (29.65%), metabolic process (28.89%) and single-organism process (16.39%) in biological processes; cell (19.17%), cell part (18.06%) and membrane (17.66%) in the cellular components; and catalytic activity (44.58%), binding (42.53%) and structural molecule activity (5.44%) in molecular functions (Additional file 5: Table S5). A total of 1551 proteins were enriched in 1927 GO terms, and the top five enriched GO terms were metabolic process, intracellular, cell, organic substance metabolic process and primary metabolic process (Fig. 5c; Additional file 6: Table S6). Among the 36 WFB-specific proteins, six, two and 14 proteins were categorized into biological processes, cellular components and molecular functions, respectively (Additional file 4: Table S4). The GO annotation information suggests that these proteins may play pivotal roles in cellular metabolism, lipid synthesis, nucleic acid metabolism, signal transduction and protein synthesis processes.

KEGG pathway annotations showed that, of all the WFB proteins, 1123 proteins were classified into five major categories and 34 subcategories (Fig. 5d; Additional file 7: Table S7). The category of translation accounted for the largest proportion at 12.30%, followed by folding, sorting and degradation (10.23%), and signal transduction (8.98%). KEGG pathway enrichment analysis revealed that the total proteins were mapped to 311 pathways, and the top five pathways included ribosome, spliceosome, RNA transport, glycolysis/gluconeogenesis and carbon metabolism. In addition, peroxisome, proteasome, protein processing in endoplasmic reticulum and fatty acid degradation were ranked in the top 30 pathways (Fig. 5e, Additional file 8: Table S8). Among the 36 WFB-specific proteins, eight were annotated by KEGG (Additional file 4: Table S4), suggesting their potential crucial roles in various biological processes, including nucleotide metabolism, lipid synthesis, protein processing and spliceosome formation.

COG database annotations showed that, of all the WFB WFB proteins, 1193 proteins were classified into 21 COG categories. The category exhibiting the greatest proportion was translation, ribosomal structure and biogenesis (20.29%), followed by posttranslational modification, protein turnover, chaperones (17.52%), general function prediction only (8.21%), energy production and conversion (7.54%), and signal transduction mechanisms (7.04%) (Fig. 5f; Additional file 9: Table S9). Among the 36 WFB-specific proteins, nine were annotated within COG categories (Additional file 4: Table S4), indicating the diverse roles of these proteins in biological processes such as cell signaling, metabolic pathways, cellular structure and post-transcriptional protein modification.

### Bioinformatic analysis of DEPs between WFBs and the oocyst wall

**Fig. 6:**
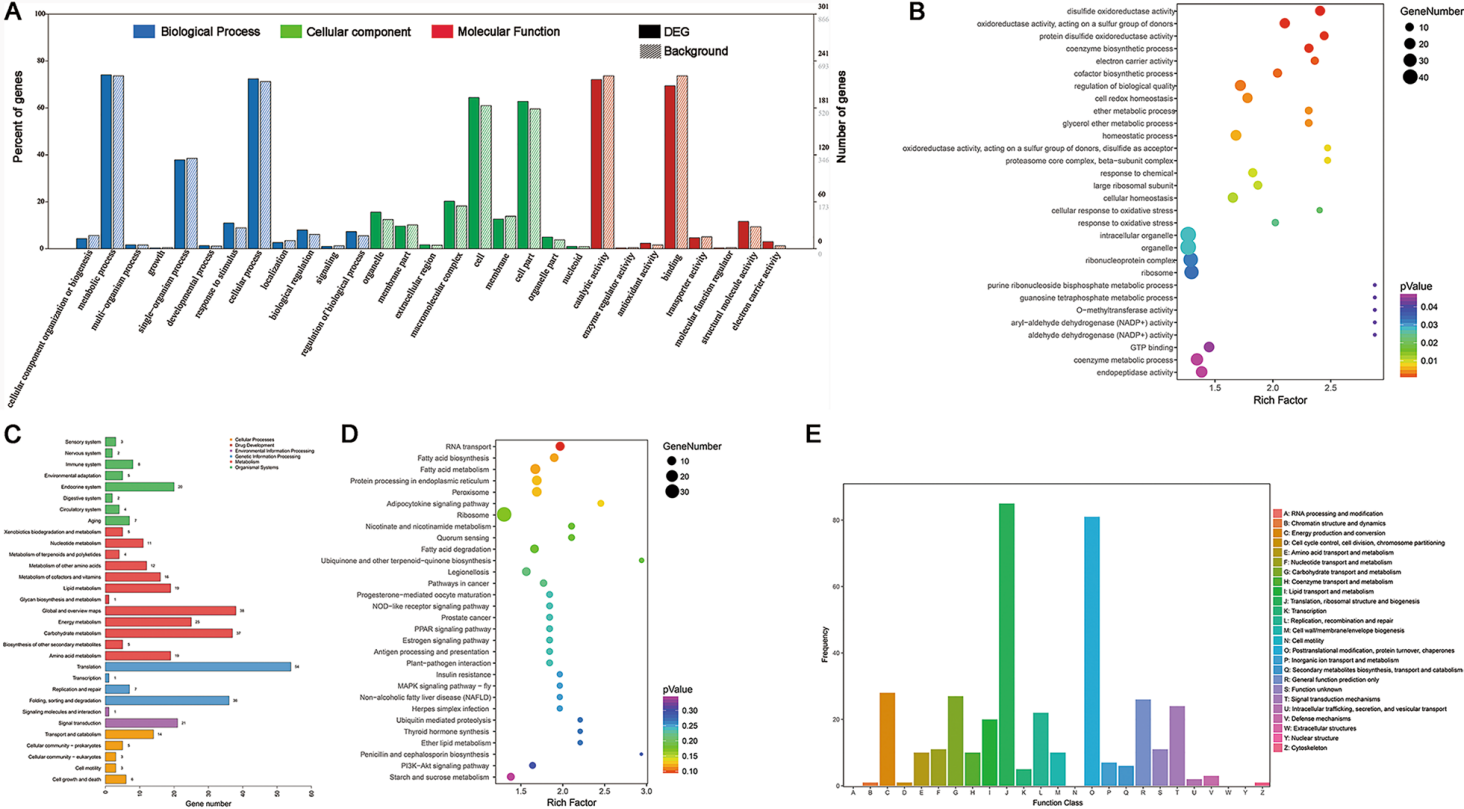
Bioinformatic analysis results of DEPs between WFBs and the OW. **a** Summary of second-level GO assignments for DEPs. The* x*-axis represents the second-level functional categories from the GO database, and the* y*-axis represents the percentage (left) and number (right) of DEPs corresponding to the genes in each second-level functional category. **b** Top 30 GO enrichment results for DEPs. The* x*-axis represents the results of Input frequency/Background frequency in the enrichment analysis, and the* y*-axis represents enriched GO terms. **c** Summary of second-level KEGG pathway analysis for DEPs. The* x*-axis represents the number of genes in each pathway, and the* y*-axis indicates the main second-level pathways. **d** Top 30 KEGG enrichment results for DEPs. The* x*-axis represents the results of Input frequency/Background frequency in the enrichment analysis, and the* y*-axis represents enriched KEGG pathway terms. **e** COG function classification of DEPs. The* x*-axis indicates the different categories of COG, and the* y*-axis indicates the frequency of different categories of COG. COG, Cluster of Orthologous Groups database; DEPs, differentially expressed proteins; GO, Gene Ontology resource; KEGG, Kyoto Encyclopedia of Genes and Genomes database; OW, oocyst wall; WFBs, wall-forming bodies

In the GO analysis, 235, 198 and 290 DEPs were assigned to the categories of biological processes (12 GO terms), cellular components (9 GO terms) and molecular function (8 GO terms), respectively (Fig. 6a; Additional file 10: Table S10). The top three GO terms were similar with these of WFB proteins, expect for the replacement of ‘membrane’ with ‘macromolecular complex’ in cellular components. The 833 DEPs were enriched in 1191 GO terms, and the top five enriched GO terms were disulfide oxidoreductase activity, oxidoreductase activity, protein disulfide oxidoreductase activity, coenzyme biosynthetic process and electron carrier activity (Fig. 6b; Additional file 11: Table S11).

KEGG pathway annotations showed that 394 DEPs were classified into five major categories and 31 subcategories (Fig. 6c; Additional file 12: Table S12). The category of translation accounted for 13.71% DEPs, followed by global and overview maps (9.64%) and carbohydrate metabolism (9.39%). KEGG pathway enrichment analysis revealed that all DEPs were mapped to 166 pathways and that the top five pathways included RNA transport, fatty acid biosynthesis, fatty acid metabolism, peroxisome and protein processing in the endoplasmic reticulum. Fatty acid degradation, ubiquinone and other terpenoid-quinone biosynthesis and ubiquitin-mediated proteolysis were also ranked in the top 30 pathways (Fig. 6d; Additional file 13: Table S13).

A total of 391 DEPs were classified into 21 COG categories, of which the top five categories were similar with these of WFB proteins, expect that ‘signal transduction mechanisms’ was replaced with ‘carbohydrate transport and metabolism.’ The categories ‘translation, ribosomal structure and biogenesis’ (20.74%) and ‘posttranslational modification, protein turnover, chaperones’ (20.72%) were also ranked in the top two among 21 COG categories (Fig. 6e; Additional file 14: Table S14).

### Analysis and identification of proteins potentially involved in oocyst wall formation

**Fig. 7:**
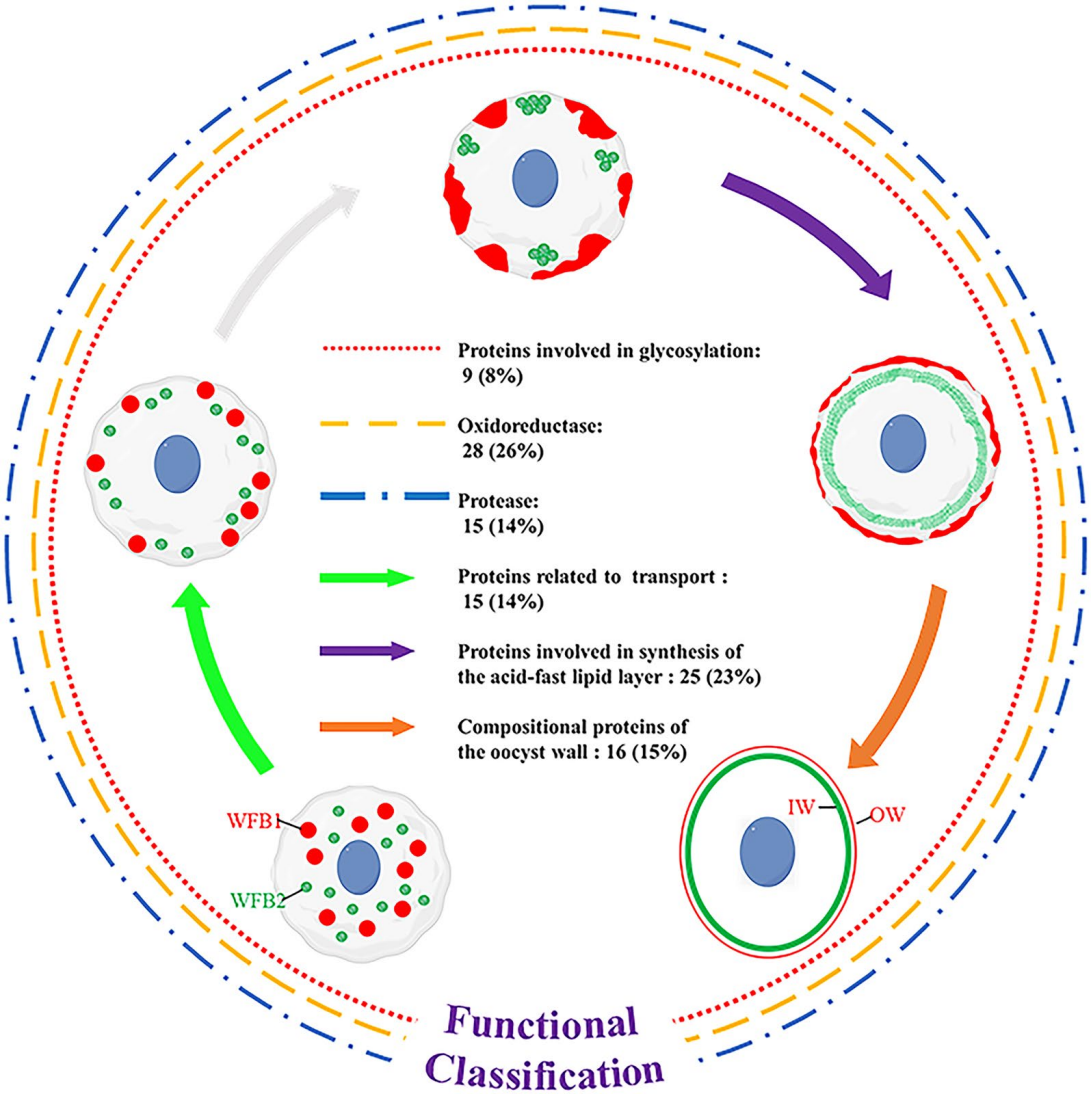
Functional classification of 108 proteins potentially involved in oocyst wall formation. Biological functions of these proteins were manually assigned based on GO, KEGG, COG, or previously published annotations. The accompanying text illustrates the number and proportion of proteins characterized by their biological functions. COG, Cluster of Orthologous Groups database; GO, Gene Ontology resource; KEGG, Kyoto Encyclopedia of Genes and Genomes database; OW, outer oocyst wall; IW, inner oocyst wall; WFB1/2, wall-forming bodies type 1/type 2

The results of the GO, KEGG and COG analyses suggest that 108 of 3059 quantified proteins are involved in oocyst wall formation. Based on their possible functions, these proteins could be classified into six distinct groups (Fig. 7; Table 2), including compositional proteins of the oocyst wall (15%), protease (14%), oxidoreductase (26%), proteins involved in glycosylation (8%), proteins involved in synthesis of the acid-fast lipid layer (23%) and proteins related to transport (14%). In addition, a total of 20 glycolytic enzymes were identified from WFBs and the oocyst wall (Additional file 15; Table S15). Subsequently, EnPDI, EnTrx and EnPGK were selected for further study.

**Table 2:** Classification of proteins involved in oocyst wall formation

Protein ID	Database	Description	Score	OW vs. WFBs (FC)	OW vs. WFBs (*P-*value)
*Compositional proteins of the oocyst wall*					
AHB64327.1	*Eimeria necatrix*	22-kDa Gametocyte protein	47.00	0.07	1.55E-02
XP_013333565.1	*Eimeria maxima*	56-kDa Gametocyte antigen, related	97.33	2.44	1.77E-02
XP_013333564.1	*Eimeria maxima*	56-kDa Gametocyte antigen, related	10.88	0.44	4.51E-01
XP_013441001.1	*Eimeria necatrix*	56-kDa Gametocyte antigen, related	482.56	0.32	2.54E-01
XP_013232286.1	*Eimeria tenella*	56-kDa Gametocyte antigen, related	301.26	0.13	1.75E-03
AKN58547.1	*Eimeria necatrix*	59-kDa Gametocyte protein	302.22	0.93	3.23E-01
XP_013441002.1	*Eimeria necatrix*	82-kDa Gametocyte antigen, related	533.72	1.36	6.90E-02
AAO47083.2	*Eimeria maxima*	82-kDa Gametocyte antigen	12.64	0.41	1.13E-02
XP_013440134.1	*Eimeria necatrix*	Oocyst wall protein, putative	89.79	0.89	4.54E-01
XP_013440596.1	*Eimeria necatrix*	Microneme protein, putative	13.96	1.59	5.59E-04
XP_013438712.1	*Eimeria necatrix*	Microneme protein, putative	20.90	0.11	3.86E-02
XP_013433151.1	*Eimeria necatrix*	Microneme protein 2, putative	11.41	0.19	6.56E-04
XP_013231935.1	*Eimeria tenella*	Microneme protein 4	15.78	0.36	5.46E-03
XP_013235137.1	*Eimeria tenella*	PAN domain-containing protein, related	12.04	0.34	3.29E-04
XP_013432524.1	*Eimeria necatrix*	PAN domain-containing protein, putative	16.07	0.33	5.25E-04
XP_013434151.1	*Eimeria necatrix*	PAN domain-containing protein, related	27.50	0.18	6.42E-03
*Protease*					
CAK51402.1	*Eimeria tenella*	Subtilisin-like	7.81	3.24	1.73E-04
XP_013433646.1	*Eimeria necatrix*	Subtilase family serine protease, putative	46.31	2.07	1.91E-03
XP_013335882.1	*Eimeria maxima*	Subtilase family serine protease, putative	3.53	1.28	2.91E-01
XP_013337377.1	*Eimeria maxima*	Subtilase family serine protease, putative, partial	16.05	0.02	3.62E-01
XP_013437838.1	*Eimeria necatrix*	Subtilase family serine protease, putative	26.72	0.16	1.94E-03
PUA83305.1	*Toxoplasma gondii*	Aminopeptidase N	6.46	0.89	6.67E-01
XP_013439539.1	*Eimeria necatrix*	Aminopeptidase N, putative	43.81	0.70	1.35E-02
CAC20154.1	*Eimeria tenella*	Aspartyl proteinase (Eimepsin)	88.78	2.23	7.30E-03
XP_013438518.1	*Eimeria necatrix*	Cystathionine beta-lyase, putative	18.76	1.01	9.40E-01
XP_013436878.1	*Eimeria necatrix*	O-acetylserine (thiol) lyase, putative	45.75	0.01	2.13E-02
XP_013437442.1	*Eimeria necatrix*	Trypsin, putative	12.62	0.77	6.79E-02
XP_013440178.1	*Eimeria necatrix*	Trypsin, putative	10.77	0.51	1.18E-03
XP_013235148.1	*Eimeria tenella*	GPI transamidase subunit PIG-U, putative, partial	1070.62	0.03	1.68E-03
XP_013439061.1	*Eimeria necatrix*	Gpi16 subunit, GPI transamidase domain-containing protein, putative	47.45	0.96	5.89E-01
XP_013234727.1	*Eimeria tenella*	Alanine dehydrogenase, putative	24.42	0.04	2.84E-02
*Oxidoreductase*					
XP_013439990.1	*Eimeria necatrix*	Oxidoreductase, putative	64.53	2.02	9.19E-05
XP_013439415.1	*Eimeria necatrix*	Oxidoreductase, putative	57.21	0.98	6.60E-01
XP_013234462.1	*Eimeria tenella*	Oxidoreductase, putative	9.32	0.91	5.83E-01
XP_013433824.1	*Eimeria necatrix*	Oxidoreductase, putative	201.77	0.11	3.86E-02
XP_013233307.1	*Eimeria tenella*	Oxidoreductase, putative	233.25	0.02	2.74E-02
XP_013440103.1	*Eimeria necatrix*	Oxidoreductase, putative	10.47	0.01	7.49E-04
XP_013233595.1	*Eimeria tenella*	Thioredoxin, putative	58.06	4.70	6.49E-03
XP_013439420.1	*Eimeria necatrix*	Thioredoxin, putative	587.42	2.74	2.87E-02
XP_013434962.1	*Eimeria necatrix*	thioredoxin, putative, partial	33.28	2.59	8.77E-03
XP_013440624.1	*Eimeria necatrix*	Thioredoxin, putative	120.79	1.85	6.91E-05
XP_013437378.1	*Eimeria necatrix*	Thioredoxin, putative	32.07	1.32	2.94E-02
XP_013436566.1	*Eimeria necatrix*	Thioredoxin domain-containing protein, putative	30.57	1.26	1.54E-01
XP_013234207.1	*Eimeria tenella*	Thioredoxin, putative	76.50	0.59	1.93E-02
XP_013433269.1	*Eimeria necatrix*	Thioredoxin, putative	73.60	0.55	2.20E-02
XP_013334736.1	*Eimeria maxima*	Thioredoxin, putative	30.69	0.24	3.30E-01
XP_013435528.1	*Eimeria necatrix*	Amiloride-sensitive amine oxidase, copper-containing, putative	615.31	1.45	1.23E-02
XP_013440644.1	*Eimeria necatrix*	Peroxiredoxin, putative	159.65	1.08	6.12E-01
XP_013335735.1	*Eimeria maxima*	Peroxiredoxin, putative	8.60	0.64	2.28E-01
XP_013439428.1	*Eimeria necatrix*	Peroxidoxin 2, putative	67.57	1.29	1.02E-01
XP_013231991.1	*Eimeria tenella*	Glutathione/thioredoxin peroxidase, putative	49.42	2.77	8.32E-06
XP_013235399.1	*Eimeria tenella*	Quinone oxidoreductase, putative	3.12	1.19	7.87E-02
XP_013440081.1	*Eimeria necatrix*	Malate:quinone oxidoreductase, putative	40.98	1.63	2.70E-03
CAK51433.1	*Eimeria tenella*	Malate:quinone oxidoreductase, putative	57.07	0.85	3.15E-01
XP_013435736.1	*Eimeria necatrix*	Glucose-methanol-choline oxidoreductase, putative	34.68	1.47	1.01E-02
XP_013435909.1	*Eimeria necatrix*	Protein disulfide isomerase, putative	389.73	1.84	3.23E-02
XP_013229611.1	*Eimeria tenella*	Protein disulfide-isomerase, putative	30.46	1.27	1.38E-01
XP_013437243.1	*Eimeria necatrix*	Protein disulfide-isomerase, putative	64.17	1.00	9.60E-01
XP_013231631.1	*Eimeria tenella*	Protein disulfide isomerase, putative	119.95	0.10	2.42E-02
*Proteins involved in glycosylation*					
XP_013435622.1	*Eimeria necatrix*	UDP-*N*-acetylglucosamine transporter, putative	12.51	0.97	5.58E-01
XP_013438147.1	*Eimeria necatrix*	UDP-*N*-acetylglucosamine pyrophosphorylase, putative	99.55	1.45	8.61E-02
ACV81910.1	*Eimeria tenella*	UDP-*N*-acetylglucosamine pyrophosphorylase, partial	3.90	1.16	3.42E-01
XP_013432573.1	*Eimeria necatrix*	UDP-*N*-acetyl-d-galactosamine:polypeptide* N*-acetylgalactosaminyltransferase T5, putative	24.08	1.74	1.00E-02
XP_013438762.1	*Eimeria necatrix*	UDP-*N*-acetyl-d-galactosamine:polypeptide* N*-acetylgalactosaminyltransferase T3, putative, partial	31.52	1.13	5.28E-02
XP_013229719.1	*Eimeria tenella*	UDP-*N*-acetyl-d-galactosamine:polypeptide* N*-acetylgalactosaminyltransferase T2, putative	60.35	1.14	1.41E-01
XP_013440716.1	*Eimeria necatrix*	UDP-glucose 4-epimerase, putative	23.04	0.74	1.22E-05
XP_013432924.1	*Eimeria necatrix*	Glucosamine–fructose-6-phosphate aminotransferase (isomerizing), putative	139.34	1.55	1.96E-02
XP_013434824.1	*Eimeria necatrix*	Dolichyl-di-phosphooligosaccharide-protein glycotransferase, putative	18.30	1.05	5.83E-01
Proteins involved in synthesis of the acid-fast lipid layer					
XP_013439980.1	*Eimeria necatrix*	Very long-chain acyl-CoA synthetase, putative	217.15	2.00	4.52E-05
XP_013433087.1	*Eimeria necatrix*	Type I fatty acid synthase, putative	363.13	2.27	2.38E-03
KFG59574.1	*Toxoplasma gondii*	Putative type I fatty acid synthase, partial	50.39	1.17	1.69E-01
XP_013438000.1	*Eimeria necatrix*	Sterol O-acyltransferase, putative	15.46	1.11	5.60E-01
XP_013233477.1	*Eimeria tenella*	Sterol O-acyltransferase, putative	12.80	0.79	4.43E-02
XP_013439400.1	*Eimeria necatrix*	Polyketide synthase, related	843.39	1.36	1.45E-02
XP_013434943.1	*Eimeria necatrix*	Polyketide synthase, related, partial	489.44	0.91	8.28E-02
XP_013440740.1	*Eimeria necatrix*	Phospholipase/carboxylesterase domain containing protein, putative	22.46	1.49	1.01E-03
XP_013228414.1	*Eimeria tenella*	Phospholipase/carboxylesterase, putative	45.28	1.11	3.91E-01
XP_013228107.1	*Eimeria tenella*	Peroxisomal multifunctional enzyme, putative	18.88	0.00	3.73E-01
XP_013228145.1	*Eimeria tenella*	Peroxisomal multifunctional enzyme type 2, putative	136.71	1.79	8.17E-03
XP_013432721.1	*Eimeria necatrix*	Peroxisomal multifunctional enzyme type 2, putative	238.79	1.12	2.08E-01
XP_013439788.1	*Eimeria necatrix*	Glycerol-3-phosphate dehydrogenase, putative	102.28	1.07	3.47E-01
XP_013438016.1	*Eimeria necatrix*	Glycerol-3-phosphate dehydrogenase, putative	97.95	1.07	1.86E-01
XP_013440415.1	*Eimeria necatrix*	Glycerol-3-phosphate acyltransferase, putative	40.04	0.86	2.70E-01
XP_013434136.1	*Eimeria necatrix*	Glycerol-3-phosphate acyltransferase, putative	68.91	0.02	3.63E-01
XP_013432854.1	*Eimeria necatrix*	Fatty acyl-CoA desaturase, putative	56.87	1.02	6.11E-01
XP_013227826.1	*Eimeria tenella*	Fatty acid elongation protein, putative	4.69	0.23	1.10E-01
XP_013435815.1	*Eimeria necatrix*	Enoyl-CoA hydratase/isomerase family protein, putative	43.00	1.23	2.60E-02
XP_013439720.1	*Eimeria necatrix*	Diacylglycerol acyltransferase family protein, related	25.65	1.13	2.66E-01
XP_013436140.1	*Eimeria necatrix*	Acyl-coenzyme A oxidase, putative	21.45	0.87	1.95E-01
XP_013229650.1	*Eimeria tenella*	Acyl-CoA-binding protein, putative	2.34	1.69	2.78E-04
XP_013234417.1	*Eimeria tenella*	acyl-CoA-binding protein, putative	12.27	0.76	3.87E-01
XP_013438170.1	*Eimeria necatrix*	Acetyl-CoA acyltransferase B, putative	25.77	1.58	5.45E-03
XP_013228517.1	*Eimeria tenella*	1-acyl-sn-glycerol-3-phosphate acyltransferase, putative	47.35	1.05	2.95E-01
*Proteins related to transport*					
AFS30550.1	*Eimeria tenella*	Dynein light chain 8a protein	6.88	0.82	2.51E-02
XP_013228555.1	*Eimeria tenella*	Dynein intermediate chain, putative	20.76	0.77	2.68E-01
XP_013436598.1	*Eimeria necatrix*	Dynein heavy chain protein, related	251.55	0.81	5.02E-02
XP_013441055.1	*Eimeria necatrix*	Dynamin-like protein, putative	36.03	3.25	1.10E-02
XP_013335061.1	*Eimeria maxima*	Dynamin-like protein, putative	52.61	1.92	9.88E-03
XP_013231245.1	*Eimeria tenella*	Dynamin-like protein, putative	65.53	1.38	7.04E-02
XP_013433064.1	*Eimeria necatrix*	Dynamin-like protein, putative	41.46	1.23	7.56E-03
XP_013440088.1	*Eimeria necatrix*	Actin-like protein 3b, putative	8.42	0.45	2.70E-03
XP_013236163.1	*Eimeria tenella*	Actin, putative	25.68	1.22	3.17E-01
XP_013229727.1	*Eimeria tenella*	Actin, putative	13.80	1.08	4.59E-01
EPR56714.1	*Toxoplasma gondii*	Actin	206.62	0.05	3.45E-03
XP_013436734.1	*Eimeria necatrix*	Actin-like family protein ARP4a, putative	15.94	1.12	2.44E-02
XP_013231284.1	*Eimeria tenella*	Actin-like family protein ARP4a, putative	20.22	0.74	7.30E-03
PIL95857.1	*Toxoplasma gondii*	Actin like protein ALP1, partial	2.37	0.85	8.38E-02
ABY64746.1	*Eimeria tenella*	Actin depolymerizing factor	70.75	1.71	9.04E-03

*FC* Fold change,* OW* oocyst wall,* WFBs* wall-forming bodies

#### Cloning and expression of EnPDI, EnTrx and EnPGK

The complementary DNA (cDNA) sequence of EnPDI protein was amplified to a length of 1473 bp (GenBank accession number: OR105511; Additional file 16: Figure S1A1) and encoded a 490-amino acid (aa) polypeptide with a 21-aa signal peptide and a predicted molecular weight of approximately 53.96 kDa; the deduced protein sequence (GenBank: WMD29389.1) was 98.6% sequence identity to the sequence deposited in the NCBI database (XP_013435909.1) (Additional file 17: Figure S2A). The cDNA sequence of EnTrx protein was amplified to a length of 1329 bp (GenBank accession number: OR105512; Additional file 16: Figure S1A2) and encoded a 442-aa polypeptide with a 38-aa signal peptide and a predicted molecular weight of approximately 49.57 kDa; the deduced protein sequence (GenBank: WMD29390.1) was 100.0% sequence identity to the sequence deposited in the NCBI database (XP_013439420.1) (Additional file 17: Figure S2B). The cDNA sequence of EnPGK protein was amplified to a length of 1206 bp (GenBank Accession number: OR105509, Additional file 16: Figure S1A3) and encoded a 401-aa polypeptide without a signal peptide and with a predicted molecular weight of approximately 42.33 kDa; the deduced protein sequence (GenBank: WMD29387.1) was 90.5% sequence identity to the sequence deposited in the NCBI database (XP_013438037.1) (Additional file 17: Figure S2C).

**Fig. 8:**
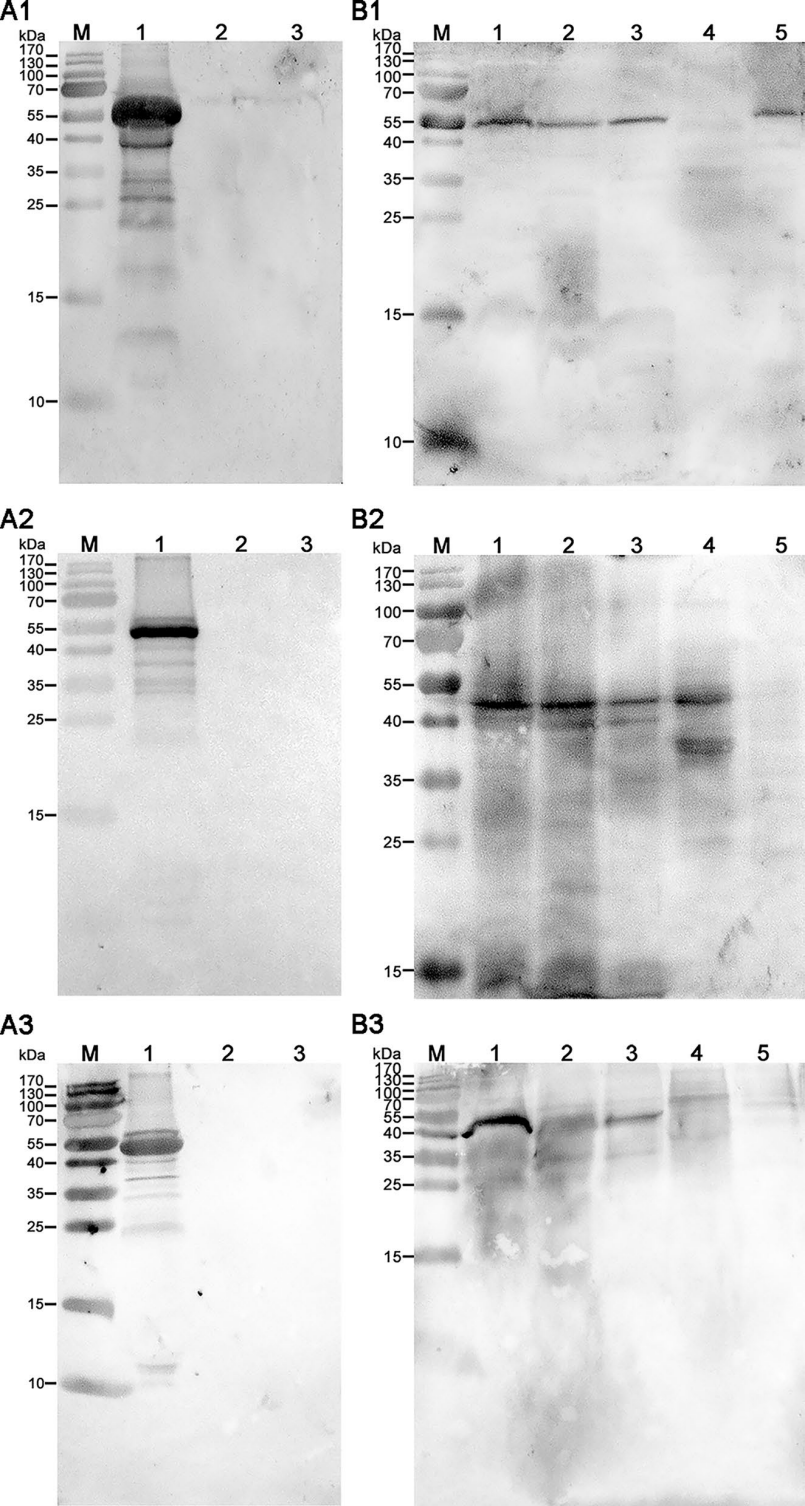
Expression and western blot analysis of rEnPDI, rEnTrx and rEnPGK.** a1**-**a3** Western blot detection of the recombinant proteins. rEnPDI (A1, approx. 57 kDa), rEnTrx (A2, approx. 52 kDa) and rEnPGK (A3, approx. 45 kDa) could be specifically recognized by the anti-6×His tag monoclonal antibody with the specific bands. Lanes: M, Protein marker; 1, BL21(DE3) cells transformed with recombinant vector and induced by 1 mM IPTG; 2, BL21 (DE3) cells transformed with the empty vector and induced by 1 mM IPTG; 3, BL21(DE3) cells induced with 1 mM IPTG.** b1**-**b3** Native proteins extracted from second-generation merozoites, third-generation merozoites, gametocytes, unsporulated oocysts and sporulated oocysts) were detected by mouse anti-rEnPDI (B1), rEnTrx (B2) and rEnPGK (B3) polyclonal antibody. Lanes: M, Protein marker; 1, second-generation merozoites; 2, third-generation merozoites; 3, gametocytes; 4, unsporulated oocysts; 5, sporulated oocysts. rEnPDI/rEnTrx/rEnPGK, Recombinant *E. necatrix* disulfide isomerase/thioredoxin/phosphoglycerate kinase

The recombinant bacteria containing expression vectors (Additional file 16: Figure S1B1-B3) were induced with IPTG. rEnPDI was expressed in soluble form, with a molecular weight of about 57 kDa, while rEnTrx and rEnPGK were expressed as inclusion bodies, with a molecular weight of about 52 and 45 kDa, respectively. The bands of the expected size (57, 52 and 45 kDa, respectively) could be detected when bacterial lysates containing the recombinant protein were probed with the anti-6×His tag monoclonal antibody (Fig. 8a1-a3).

#### Detection of native EnPDI, EnTrx and EnPGK

The native EnPDI protein (approx. 55 kDa) was detected by the anti-EnPDI pAb in the MZ-2, MZ-3, GAM and SO (Fig. 8b1). The native EnTrx protein (approx. 50 kDa) was detected by the anti-EnTrx pAb in the MZ-2, MZ-3, GAM and UO (Fig. 8b2). In addition, a band of approximately 39 kDa presented in MZ-3 and GAM, and a band of approximately 37 kDa presented in UO. The native EnPGK protein (approx. 45 kDa) was detected by the anti-EnPGK pAb in MZ-2, MZ-3 and GAM (Fig. 8B3).

#### Localization of EnPDI, EnTrx and EnPGK in GAM and UO

**Fig. 9:**
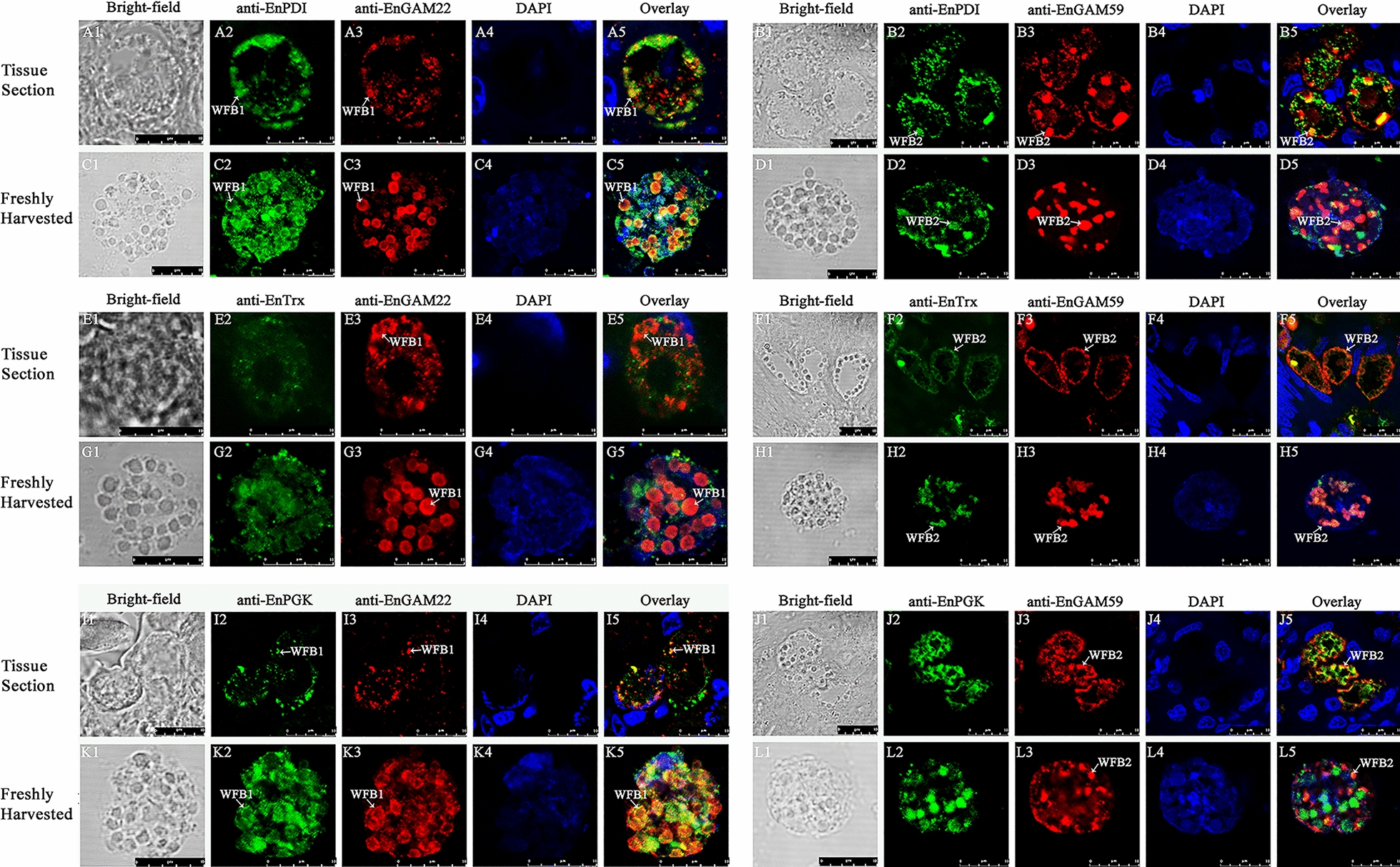
Immunofluorescence analysis of macrogametes in tissue sections (**a**, **b**, **e**, **f**, **i**, **j**) and in freshly harvested (**c**, **d**, **g**, **h**, **k**, **l**) of *E. necatrix*. Immunolabeled with mouse anti-rEnPDI (**a**-**d**), mouse anti-rEnTrx (**e**–**h)** and mouse anti-rEnPGK (**i**-**l**). Anti-rEnGAM22 rabbit sera (**a**, **c**, **e**, **g**, **i**, **k**) served as counterstaining of WFB1s, and anti-rEnGAM59 rabbit sera (**b**, **d**, **f**, **h**, **j**, **l**) served as counterstaining of WFB2s. Scale bar: 10 μm. Anti-rEnGAM22/-rENGAM59, Anti-recombinant* E. necatrix* GAM protein 22/GAM protein 59; rEnPDI/rEnTrx/rEnPGK, recombinant *E. necatrix* protein disulfide isomerase/thioredoxin/phosphoglycerate kinase; WFB1/2, wall-forming body type 1/type 2

**Fig. 10:**
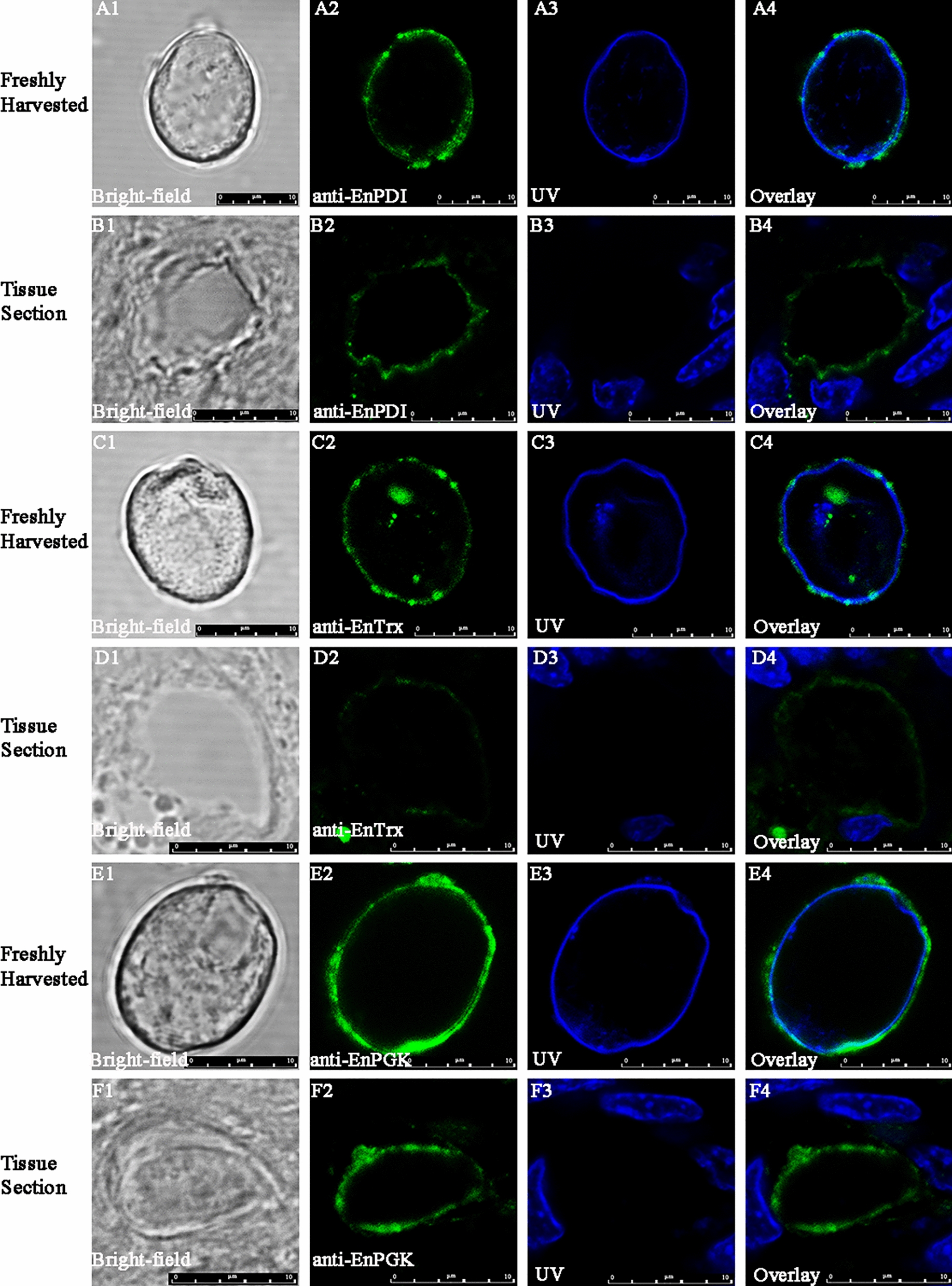
Immunofluorescence microscopy analysis of unsporulated oocysts freshly harvested (**a**, **c**, **e**) and in tissue section (**b**, **d**, **f**) of *E. necatrix*. Utilizing mouse anti-rEnPDI (**a**, **b**), mouse anti-rEnTrx (**c**, **d**) and mouse anti-rEnTrx (**e**, **g**) with DAPI counterstaining (**a3**, **b3**, **c3**, **e3**, **f3**). Scale bar: 10 μm, rEnPDI/rEnTrx/rEnPGK, recombinant *E. necatrix* protein disulfide isomerase/thioredoxin/phosphoglycerate kinase

Immunolocalization analysis revealed that EnPDI, EnTrx and EnPGK possessed distinct localization patterns in the macrogametes of *E. necatrix*. The anti-rEnPDI pAb was co-localized to WFB1s with anti-rEnGAM22 pAb (Fig. 9a1–a5, c1–c5) and to WFB2s with anti-rEnGAM59 pAb (Fig. 9b1-b5, d1-d5), suggesting that EnPDI presented in both types of WFBs. In comparison, anti-rEnTrx pAb was co-localized to WFB2s with anti-rEnGAM59 pAb (Fig. 9f1-f5, h1-h5) but not to WFB1s with anti-rEnGAM22 pAb (Fig. 9e1-e5, g1-g5), indicating that EnTrx presented only in WFB2s. In contrast, anti-rEnPGK pAb was co-localized to WFB1s with anti-rEnGAM22 pAb (Fig. 9i1-i5, k1-k5) but not to WFB2s with anti-rEnGAM59 pAb (Fig. 9J1-J5, L1-L5), indicating that EnPGK presented only in WFB1s. Surprisingly, IFA with anti-rEnPDI, rEnTrx and rEnPGK pAbs showed that these proteins were also localized to the outer layer of the oocyst wall (Fig. 10a-b, c-d and e–f, respectively). No reaction was detected when negative control serum was used (Additional file 18: Figure S3; Additional file 19: Figure S4).

.

## Discussion

Based on the results of this study: (i) two tyrosine-rich precursor proteins (gam56 and gam82) were proteolytically processed into smaller peptides that were incorporated into the developing oocyst wall of *E. maxima*; (ii) oocysts gave a characteristic blue autofluorescence at 340–360 nm; (iii) dityrosine and 3,4-dihydroxyphenylalanine (DOPA) were biochemically detectable in oocyst extracts; and (iv) peroxidase activity was detectable in the WFBs of macrogametes. Belli et al. proposed a model for the wall formation of coccidian oocyst [5]: large tyrosine-rich proteins or scaffolding proproteins are synthesized and stockpiled in WFBs of macrogametes, and at a certain developmental time point, the proproteins are processed to smaller, tyrosine-rich proteins; the tyrosine residues in the proteins undergo oxidative phenolic coupling by an enzyme, such as peroxidase, which leads to the formation of protein–dityrosine, dehydration and oocyst wall hardening. Belli et al. also suggested that the molecular machinery involved in the assembly of the oocyst wall, including precursor proteins, processing enzymes, cross-linking enzymes and cofactors, were housed in the WFBs in macrogametocytes [5]. However, the exact contents of WFBs remain unclear to date. In the present study, we applied TMT labeling coupled with LC–MS/MS to profile and compare the proteomes of *E. necatrix* WFBs and the oocyst wall. A total of 3009 proteins were identified in the WFBs, with 108 identified proteins proposed to be involved in oocyst wall formation. Based on their possible functions, these proteins were classified into six distinct groups: compositional proteins of the oocyst wall, protease, oxidoreductase, proteins involved in glycosylation, proteins involved in synthesis of the acid-fast lipid layer and proteins related to transport. To the best of our knowledge, this is the first report on the proteomes of WFBs and the oocyst wall of *E. necatrix*.

### Compositional proteins of the oocyst wall

Three groups of gametocyte proteins have been confirmed to participate in oocyst wall formation. One group consists of tyrosine-rich glycoproteins that localize to WFB2 of macrogametocytes and the inner wall of *Eimeria* oocysts, including Gam82, Gam56 and Gam59 [8, 26]. Another group is histidine-proline-rich protein that localizes to WFB1 of macrogametocytes and the outer wall of *Eimeria* oocyst, with Gam22 being a prominent representative of this group [15]. The third group is cysteine-rich proteins (namely oocyst wall protein [OWP]) that localize to WFB1 and the inner wall of *Cryptosporidium* oocyst [27], and to WFB1 and the outer wall of *Eimeria nieschulzi* oocyst [28]. In the present study, Gam82 (XP_013441002.1, AAO47083.2), Gam56 (XP_013333565.1, XP_013333564.1, XP_013441001.1, XP_013232286.1), Gam59 (AKN58547.1), Gam22 (AHB64327.1) and OWP (XP_013440134.1) were identified in both the WFBs and the oocyst wall of *E. necatrix*. Of these proteins, Gam56, Gam59 and Gam22 have been identified and found to be involved in formation of the inner and outer wall of the *E. necatrix* oocyst [15, 26], whereas the functions of Gam82 and OWP need to be confirmed.

Ferguson et al. found that the microneme protein MIC4 or an MIC4-like protein was expressed within the WFB1a of the macrogamete and associated with oocyst wall formation in *T. gondii* [29]. Proteomic analysis of fractionated *Toxoplasma* oocysts revealed the distinct abundance of PAN domain-containing proteins that are characterized by a disulfide bridge folding pattern [19]. The transcripts for PAN domain-containing proteins also exhibited upregulation in *E. tenella* gametocytes [30]. In the present study, four MIC proteins (XP_013440596.1, XP_013438712.1, XP_013433151.1, XP_013231935.1) and three PAN domain-containing proteins (XP_013235137.1, XP_013432524.1, XP_013434151.1) were identified. However, further study is required to determine the precise roles of these proteins in oocyst wall formation.

### Proteases and oxidoreductase

Proteases are responsible for cleaving precursor proteins such as GAM56 and GAM82 into smaller peptides, and oxidoreductases create dityrosine bonds and disulfide bridges to establish a sturdy matrix. Therefore, these two types of enzymes are crucial in terms of comprehending the mechanisms governing oocyst wall formation. In this study, several proteases, including one subtilisin-like protein (CAK51402.1), four subtilase family serine proteases (XP_013433646.1, XP_013335882.1, XP_013337377.1, XP_013437838.1), two aminopeptidase N proteins (PUA83305.1, XP_013439539.1), one aspartyl proteinase (Eimepsin, CAC20154.1) and several oxidoreductases, including six oxidoreductases (XP_013439990.1, XP_013439415.1, XP_013234462.1, XP_013433824.1, XP_013233307.1, XP_013440103.1), nine thioredoxins (XP_013233595.1, XP_013439420.1, XP_013434962.1, XP_013440624.1, XP_013437378.1, XP_013436566.1, XP_013234207.1, XP_013433269.1, XP_013334736.1), one amiloride-sensitive copper-containing amine oxidase (XP_013435528.1) and three peroxiredoxins (XP_013440644.1, XP_013335735.1, XP_013439428.1), were identified. Similarly, the transcripts encoding these proteases and oxidoreductases were upregulated in *E. tenella* or *E. necatrix* gametocytes [30, 31].

We also detected one cystathionine beta-lyase (XP_013438518.1), one* O*-acetylserine (thiol) lyase (XP_013436878.1), two trypsin (XP_013437442.1, XP_013440178.1), two GPI transamidase subunits (XP_013235148.1, XP_013439061.1), one putative alanine dehydrogenase (XP_013234727.1), one glutathione/thioredoxin peroxidase (XP_013231991.1), three quinone oxidoreductases (XP_013235399.1, XP_013440081.1, CAK51433.1), one glucose-methanol-choline oxidoreductase (XP_013435736.1) and four protein disulfide isomerases (XP_013435909.1, XP_013229611.1, XP_013437243.1, XP_013231631.1) in this study. Of these enzymes, the cystathionine beta-lyase and* O*-acetylserine (thiol) lyase are considered to be integral to the biosynthesis of cysteine within the oocyst of *T. gondii* [32]; the transcripts encoding the two trypsin are upregulated in the *E. tenella* gametocyte [33]; the GPI transamidase subunits and alanine dehydrogenase have been identified in *T. gondii* oocyst wall proteins [19]; the glutathione/thioredoxin peroxidase is thought to be responsible for catalyzing oxidation using H_2_O_2_ to produce dityrosine cross-links between oocyst wall proteins of *E. tenella* [34]; the transcript level for quinone oxidoreductase exhibits upregulation in *E. tenella* gametocyte[34]; the glucose-methanol-choline oxidoreductase has been confirmed to be localized on the WFBs in *E. necatrix* gametocytes [35]; and the PDIs exhibit transglutaminase activity in *Giardia*, catalyzing the formation of disulfide bond and isopeptide protein crosslinks in vivo and in vitro, thereby contributing significantly to cyst wall formation [10]. Therefore, it can be inferred that these identified enzymes are likely to play pivotal roles in oocyst wall formation of *E. necatrix*.

PDI and Trxs belong to the thioredoxin superfamily, whose members share a common structural motif named the thioredoxin fold. They are involved in disulfide oxidoreduction and/or isomerization [11]. Previous studies revealed that* Cryptosporidium *oocyst wall proteins (COWPs) and their orthologs in *T. gondii* participate in the wall formation of oocysts [36, 37], and that these cysteine motif-containing proteins are cross-linked via disulfide bonds between the cysteine residues [27, 36]. A recent study showed that EnOWP13 is a protein specifically localized to WFBI and a component of the outer oocyst wall of *E. nieschulzi*, with the results suggesting the existence of isopeptide bonds in the oocyst wall that were regularly stimulated by transglutaminase activity [28]. In the present study, one oocyst wall protein (OWP), four PDIs and nine Trxs were detected, of which EnPDI (XP_013435909.1) and EnTrx (XP_013439420.1) were chosen for further study. The native EnPDI protein (approx. 55 kDa) was detected in MZ-2, MZ-3, GAM and SO, and was localized in WFB1s and WFB2s. The native EnTrx protein (approx. 50 kDa) was present in MZ-2, MZ-3, GAM and UO, and was localized in WFB2s rather than WFB1s. However, both EnPDI and EnTrx were localized in the outer layer of the oocyst wall. These results implied that apart from via dityrosine bonds catalyzed by peroxidase, the oocyst wall proteins of *Eimeria* (such as OWP) may undergo cross-linking via isopeptide bonds or disulfide bonds catalyzed by PDIs or Trxs, ultimately contributing to the rigidification of the oocyst wall. The precise roles of EnPDI and EnTrx in oocyst wall formation still has to be revealed, and whether the OWP (XP_013440134.1) participates in oocyst wall formation of *E. necatrix* is a question for future research.

### Proteins involved in glycosylation

Glycoproteins like GAM56 and GAM82 play a pivotal role in oocyst wall composition, characterized by their extensive glycosylation, which is closely associated with a co-regulated glycosylation pathway [6, 9, 38]. In the present study, we identified nine proteins associated with the glycosylation pathway: one UDP-*N*-acetylglucosamine transporter (XP_013435622.1); two UDP-*N*-acetylglucosamine pyrophosphorylases (UAPs; XP_013438147.1, ACV81910.1); three UDP-*N*-acetyl-d-galactosamines (polypeptide* N*-acetylgalactosaminyl transferase (GalNAc-T): XP_013432573.1, XP_013438762.1, XP_013229719.1); one UDP-glucose 4-epimerase (UGE; XP_013440716.1); one glucosamine-fructose-6-phosphate aminotransferase (GFAT; isomerizing; XP_013432924.1) and one dolichyl-diphosphooligosaccharide-protein glycotransferase (XP_013434824.1). Correspondingly, transcripts for these glycosylation-related proteins have been found to be upregulated in *E. tenella* and *E. necatrix* gametocytes [30, 31]. The results suggested that these identified proteins likely play roles in oocyst wall glycosylation during *E. necatrix* oocyst wall formation.

### Proteins involved in synthesis of the acid-fast lipid layer

A previous study showed that the abundant neutral lipids such as cholesterol and triglycerides in WFB1, which align at the periphery of gametocytes and release their contents as “rafts”, contribute to the formation of the patchwork pattern in the outer oocyst wall [39]. The acid-fast stains, which typically adhere to lipids in mycobacterial cell walls, have also been observed on the oocyst wall [40]. These findings suggested that coccidia build a waxy coat of acid-fast lipids in the oocyst wall that makes them resistant to environmental stress [40]. In the present study, we identified 25 proteins that might be involved in synthesizing the acid-fast lipid layer, including one very long-chain acyl-CoA synthetase (XP_013439980.1), two type I fatty acid synthases (XP_013433087.1, KFG59574.1), two sterol* O*-acyltransferases (XP_013438000.1, XP_013233477.1), two polyketide synthases (XP_013439400.1, XP_013434943.1), two phospholipase/carboxylesterases (XP_013440740.1, XP_013228414.1), three peroxisomal multifunctional enzymes (XP_013228107.1, XP_013228145.1, XP_013432721.1) and four glycerol-3-phosphate dehydrogenases (XP_013439788.1, XP_013438016.1, XP_013440415.1, XP_013434136.1). Similarly, the transcripts encoding these proteins associated with acid-fast lipid layer synthesis have been found to be upregulated in the gametocytes of *E. tenella* and *E. necatrix* [30, 31]. However, the precise roles of these proteins in the development of the lipid layer still needs to be determined.

### Proteins related to transport

The formation of the bilayered oocyst wall is dependent on the sequential release of contents from WFB1s and WFB2s, which is facilitated by the involvement of actin and dynamin-like proteins in the transport process [31, 41]. In the present study, 15 transport-related proteins, including eight actin-like proteins (XP_013440088.1, XP_013236163.1, XP_013229727.1, EPR56714.1, XP_013436734.1, XP_013231284.1, PIL95857.1, ABY64746.1) and seven dynamin-like proteins (AFS30550.1, XP_013228555.1, XP_013436598.1, XP_013441055.1, XP_013335061.1, XP_013231245.1, XP_013433064.1), were identified. Whether these actin and dynamin-like proteins are involved in the transport and release of WFBs requires further research.

### Glycolytic enzymes identified from WFBs and oocyst wall

Interestingly, a total of 20 glycolytic enzymes were identified from WFBs and the oocyst wall, of which the PGK (XP_013438037.1) exhibited higher levels of expression in the WFBs than in the oocyst wall of *E. necatrix*. Further research revealed that the EnPGK is present in MZ-2, MZ-3 and GAM, and that it is localized in WFB1s and the outer layer of the oocyst wall but not in WFB2s. A previous study confirmed that PGK is a bona fide cell wall protein of *C. albicans* [12]. In *S. mansoni*, the subcellular localization of glycolytic enzymes directly beneath the tegument surface suggests the potential involvement of these enzymes in utilizing incoming glucose for ATP production, which is crucial for various physiological processes, such as nutrient and solute uptake, as well as cytoskeletal rearrangements [13]. How these glycolytic enzymes participate in the formation of oocyst wall still needs to be elucidated.

## Conclusions

In summary, our research represents the first attempt to investigate the protein abundance in WFBs and the oocyst wall of *E. necatrix* using TMT peptide labeling coupled with the LC–MS/MS quantitative proteomics technique. A total of 3009 and 2973 proteins were identified from WFBs and the oocyst wall of *E. necatrix*, respectively. A total of 108 proteins were proposed to be involved in oocyst wall formation, including compositional proteins of the oocyst wall, protease, oxidoreductase, proteins involved in glycosylation, proteins involved in the synthesis of the acid-fast lipid layer and proteins related to transport. Furthermore, we confirmed that EnPDI, EnTrx and EnPGK participated in the formation of the oocyst wall. While further functional studies are needed to fully elucidate the roles of these proteins in the formation of the oocyst wall, our results provide new insights into the molecular mechanisms underlying the formation of oocyst wall of *Eimeria* parasites. Moreover, our work can help in the development of novel therapeutic agents and vaccines aimed at combating coccidian transmission.

### Supplementary Information

**Additional file 1: Table S1.** WFB-specific proteins information identified between WFBs and oocyst wall groups of *Eimeria necatrix* by TMT-based quantitative proteomics.

A**dditional file 2:** **Table S2.** Differentially expressed proteins information identified between WFBs and oocyst wall groups of *Eimeria necatrix* by TMT-based quantitative proteomics.

**Additional file 3:** **Table S3.** Reliability analysis of proteomics data by Simple Western analysis (WFBs/OW).

**Additional file 4: Table S4. **Annotation of WFB-specific proteins using GO, KEGG, and COG databases.

**Additional file 5:** **Table S5.** Enrichment analysis of second-level Gene Ontology terms for the proteins identified in both WFBs and oocyst wall.

**Additional file 6: Table S6.** Enrichment analysis of Gene Ontology terms for the proteins identified in both WFBs and oocyst wall.

**Additional file 7: Table S7.** Enrichment analysis of second-level KEGG Pathway terms for the proteins identified in both WFBs and oocyst wall.

**Additional file 8: Table S8.** Enrichment analysis of KEGG Pathway terms for the proteins identified in both WFBs and oocyst wall.

**Additional file 9: Table S9.** Enrichment analysis of COG terms for the proteins identified in both WFBs and oocyst wall.

**Additional file 10:** **Table S10.** Enrichment analysis of second-level Gene Ontology terms for differentially expressed proteins between WFBs and oocyst wall.

**Additional file 11:** **Table S11.** Enrichment analysis of Gene Ontology terms for differentially expressed proteins between WFBs and oocyst wall.

**Additional file 12: Table S12.** Enrichment analysis of second-level KEGG Pathway terms for differentially expressed proteins between WFBs and oocyst wall.

**Additional file 13:** **Table S13.** Enrichment analysis of KEGG Pathway terms for differentially expressed proteins between WFBs and oocyst wall.

**Additional file 14: Table S14.** Enrichment analysis of COG terms for differentially expressed proteins between WFBs and oocyst wall.

**Additional file 15:Table S15.** Glycolytic enzymes identified from WFBs and oocyst wall.

**Additional file 16: Figure S1. A1-A3**) RT-PCR product of En*PDI* (A1), En*Trx* (A2) and En*PGK* (A3). M, *Trans*5K DNA marker (TransGen, Beijing, China); Lane1-3, the RT-PCR product.** B1-B3** Identification of recombinant prokaryotic plasmids.** B1** pET28a(+)-En*PDI* digested by *Bam*HI and *Xho*I;** B2** pET28a(+)-En*Trx* digested by *Bam*HI and *Eco*RI;** B3** pET28a(+)-En*PGK* digested by *Bam*HI and* Not*I. M, *Trans*5K DNA marker (TransGen, Beijing, China); Lane1-3, the results of restriction enzyme digestion.

**Additional file 17:** **Figure S2.** Alignment of the deduced amino acid sequence of *EnPDI* (**B1**), *EnTrx* (**B2**) and *EnPGK* (**B3**) with the sequence deposited in the NCBI database. The alignment was generated using the CLUSTALW algorithm and Lasergene software (DNASTAR), red shading corresponds to dissimilar amino acid residues.

**Additional file 18: Figure S3.** Negative control for immunofluorescence analysis of macrogametes freshly harvested (**A**) and in tissue section (**B**) of *E. necatrix*. NMS: normal mouse serum; NRS: normal rabbit serum.

**Additional file 19: Figure S4.** Negative control for immunofluorescence analysis of unsporulated oocysts freshly harvested (**A**) and in tissue section (**B**) of *E. necatrix*. NMS: normal mouse serum.

## Data Availability

The data supporting the findings of the study must be available within the article and/or its supplementary materials, or deposited in a publicly available database.

## Abbreviations

BSA: Bovine serum albumin
COG: Cluster of orthologous groups
DEPs: Differentially expressed proteins
GAM: Gametocytes
GO: Gene Ontology
HPLC: High-performance liquid chromatography
IFA: Indirect immunofluorescence analyses
KEGG: Kyoto encyclopedia of genes and genomes
LC–MS/MS: Liquid chromatography tandem-mass spectrometry
MZ-2: Second-generation merozoites
MZ-3: Third-generation merozoites
PBS: Phosphate-buffered saline
PDI: Protein disulfide isomerase
PGK: Phosphoglycerate kinase
SDS–PAGE: Sodium dodecyl sulphate–polyacrylamide gel electrophoresis
SO: Sporulated oocyst
TMT: Tandem mass tag
TNE: Tris-NaCl-EDTA
Trx: Thioredoxin
UO: Unsporulated oocyst
WFBs: Wall-forming bodies
