# Supplementary material for: Comparative proteomic analysis of wall-forming bodies and oocyst wall reveals the molecular basis underlying oocyst wall formation in Eimeria necatrix
Source: Parasit Vectors. 2023 Dec 18;16:460. doi: 10.1186/s13071-023-06076-6 (PMC10729351; doi:10.1186/s13071-023-06076-6)
Supplement: Supplementary file 3 — Additional file 3: Table S3. Reliability analysis of proteomics data by Simple Western analysis (WFBs/OW). [file 13071_2023_6076_MOESM3_ESM.docx]

**Table S3** Reliability analysis of proteomics data by Simple Western analysis (WFBs/OW)

| Accession | Description | Score | Ratio (TMT) | Grey values ratio  (Simple Western) |
| --- | --- | --- | --- | --- |
| AHB64327.1 | 22 kDa gametocyte protein [Eimeria necatrix] | 47.00 | 14.97 | >20 |
| AKN58547.1 | 59 kDa gametocyte protein [Eimeria necatrix] | 302.22 | 1.07 | 1.88 |
