# Supplementary material for: Comparative proteomic analysis of wall-forming bodies and oocyst wall reveals the molecular basis underlying oocyst wall formation in Eimeria necatrix
Source: Parasit Vectors. 2023 Dec 18;16:460. doi: 10.1186/s13071-023-06076-6 (PMC10729351; doi:10.1186/s13071-023-06076-6)
Supplement: Supplementary file 15 — Additional file 15:Table S15. Glycolytic enzymes identified from WFBs and oocyst wall. [file 13071_2023_6076_MOESM15_ESM.docx]

**Table S15** Glycolytic enzymes identified from WFBs and oocyst wall

| Protein ID | Database | Description | Score | OW-vs-WFBs. FC | OW-vs-WFBs. Pvalue |
| --- | --- | --- | --- | --- | --- |
| XP_013437914.1 | *Eimeria necatrix* | triosephosphate isomerase, putative | 20.97 | 1.35 | 1.49E-01 |
| XP_013434223.1 | *Eimeria necatrix* | triosephosphate isomerase, putative | 30.85 | 1.13 | 1.25E-01 |
| XP_013435489.1 | *Eimeria necatrix* | pyruvate kinase, putative | 454.09 | 0.68 | 3.31E-02 |
| XP_013436000.1 | *Eimeria necatrix* | phosphoglycerate mutase family protein, putative, partial | 2.72 | 1.86 | 8.10E-04 |
| XP_013440682.1 | *Eimeria necatrix* | phosphoglycerate mutase protein, putative | 4.10 | 1.22 | 7.12E-02 |
| XP_013235424.1 | *Eimeria tenella* | phosphoglycerate mutase 1, putative | 94.51 | 1.01 | 9.54E-01 |
| XP_013332747.1 | *Eimeria maxima* | phosphoglycerate kinase, putative | 31.03 | 1.64 | 1.92E-02 |
| XP_013228897.1 | *Eimeria tenella* | phosphoglycerate kinase | 119.13 | 0.94 | 6.58E-01 |
| XP_013438037.1 | *Eimeria necatrix* | phosphoglycerate kinase, putative | 165.21 | 0.54 | 5.79E-03 |
| XP_001352096.1 | *Plasmodium falciparum* | phosphoglycerate kinase | 10.09 | 0.01 | 3.66E-01 |
| XP_013438810.1 | *Eimeria necatrix* | phosphoglycerate kinase, putative | 13.47 | 1.91 | 3.65E-04 |
| XP_013434970.1 | *Eimeria necatrix* | phosphofructokinase, putative | 126.03 | 0.67 | 1.07E-02 |
| XP_013438173.1 | *Eimeria necatrix* | phosphofructokinase, putative | 232.69 | 0.63 | 9.29E-02 |
| XP_013438650.1 | *Eimeria necatrix* | hexokinase, putative, partial | 85.17 | 1.22 | 2.16E-01 |
| ANF07129.1 | *Eimeria tenella* | hexokinase | 48.96 | 0.95 | 7.22E-01 |
| XP_013440751.1 | *Eimeria necatrix* | glyceraldehyde-3-phosphate dehydrogenase, putative | 110.11 | 1.07 | 4.78E-01 |
| XP_013439292.1 | *Eimeria necatrix* | glyceraldehyde-3-phosphate dehydrogenase, putative | 282.13 | 0.79 | 2.67E-01 |
| XP_013229416.1 | *Eimeria tenella* | glyceraldehyde-3-phosphate dehydrogenase, putative | 81.39 | 0.11 | 2.27E-04 |
| XP_013437064.1 | *Eimeria necatrix* | Fructose-bisphosphate aldolase, related | 313.38 | 0.50 | 1.41E-01 |
| XP_013233987.1 | *Eimeria tenella* | Fructose-bisphosphate aldolase, related | 60.89 | 0.12 | 7.89E-03 |
