# Supplementary figures and images for: Comparative proteomic analysis of wall-forming bodies and oocyst wall reveals the molecular basis underlying oocyst wall formation in Eimeria necatrix
Source: Parasit Vectors. 2023 Dec 18;16:460. doi: 10.1186/s13071-023-06076-6 (PMC10729351; doi:10.1186/s13071-023-06076-6)

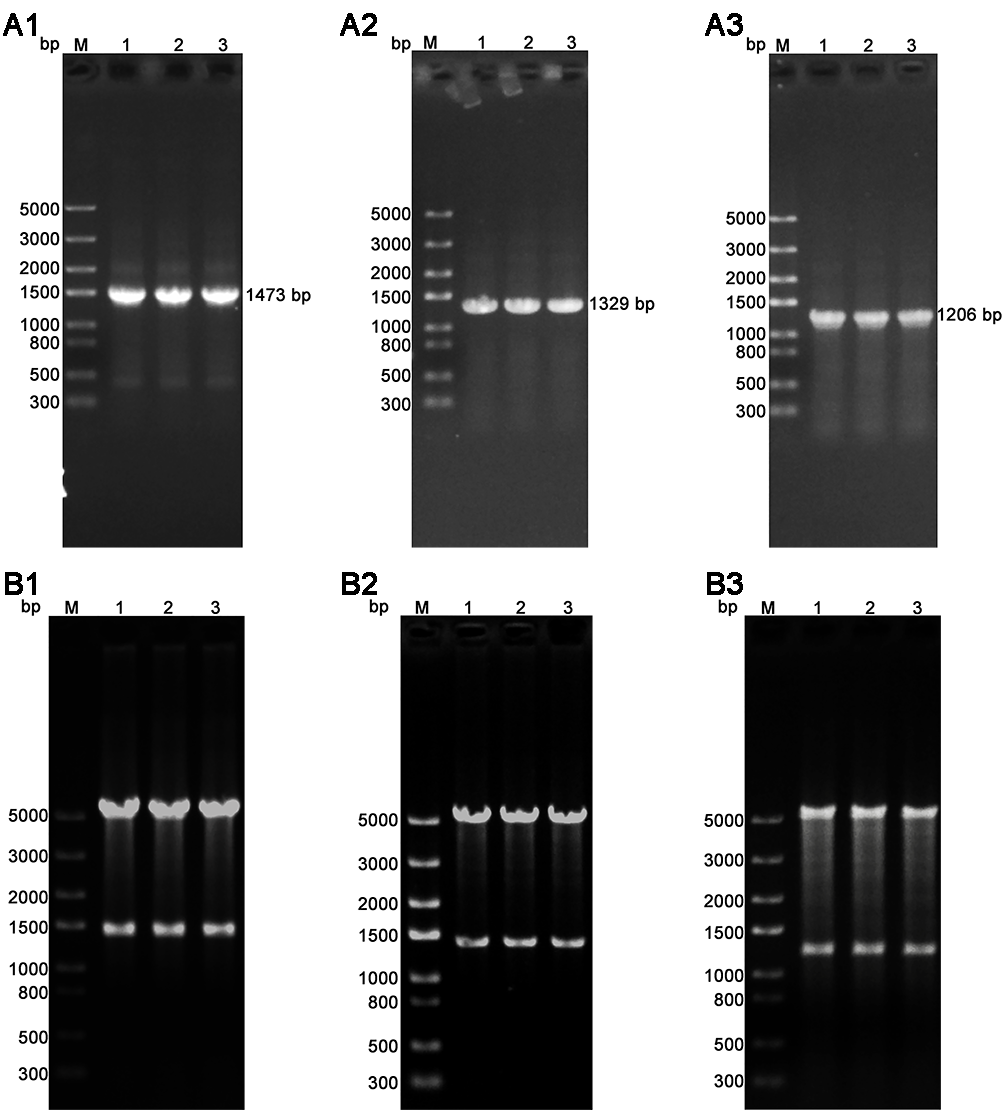

Supplement: Supplementary file 16 — Additional file 16: Figure S1. A1-A3) RT-PCR product of EnPDI (A1), EnTrx (A2) and EnPGK (A3). M, Trans5K DNA marker (TransGen, Beijing, China); Lane1-3, the RT-PCR product. B1-B3 Identification of recombinant prokaryotic plasmids. B1 pET28a(+)-EnPDI digested by BamHI and XhoI; B2 pET28a(+)-EnTrx digested by BamHI and EcoRI; B3 pET28a(+)-EnPGK digested by BamHI and NotI. M, Trans5K DNA marker (TransGen, Beijing, China); Lane1-3, the results of restriction enzyme digestion. [file 13071_2023_6076_MOESM16_ESM.tif]

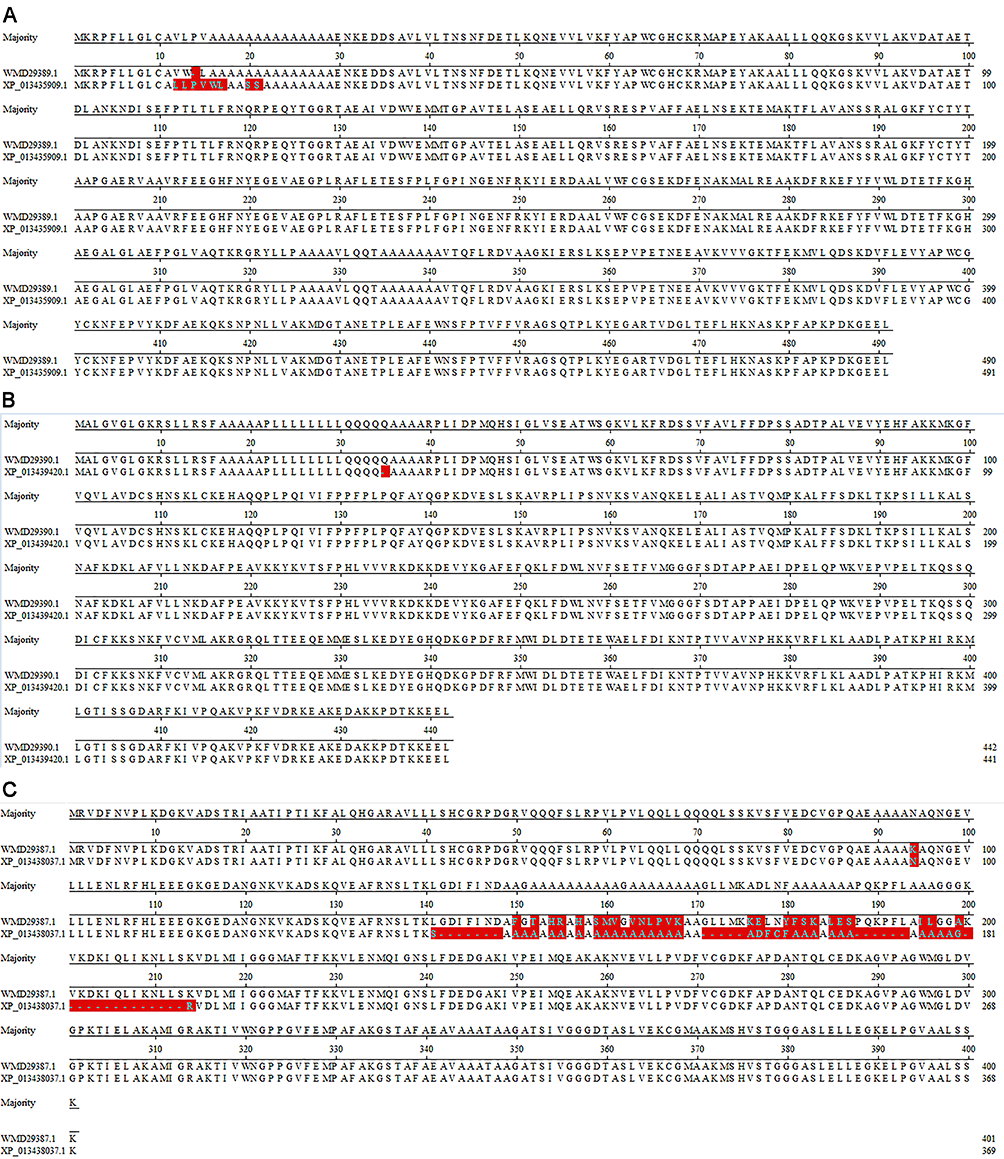

Supplement: Supplementary file 17 — Additional file 17: Figure S2. Alignment of the deduced amino acid sequence of EnPDI (B1), EnTrx (B2) and EnPGK (B3) with the sequence deposited in the NCBI database. The alignment was generated using the CLUSTALW algorithm and Lasergene software (DNASTAR), red shading corresponds to dissimilar amino acid residues. [file 13071_2023_6076_MOESM17_ESM.tif]

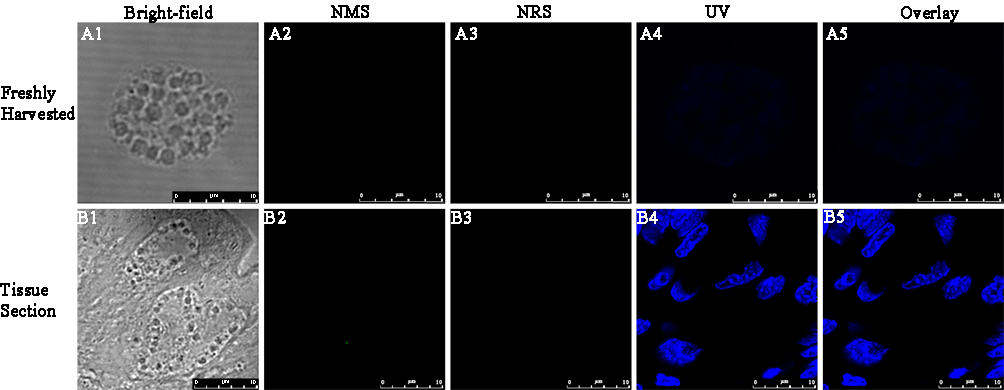

Supplement: Supplementary file 18 — Additional file 18: Figure S3. Negative control for immunofluorescence analysis of macrogametes freshly harvested (A) and in tissue section (B) of E. necatrix. NMS: normal mouse serum; NRS: normal rabbit serum. [file 13071_2023_6076_MOESM18_ESM.tif]

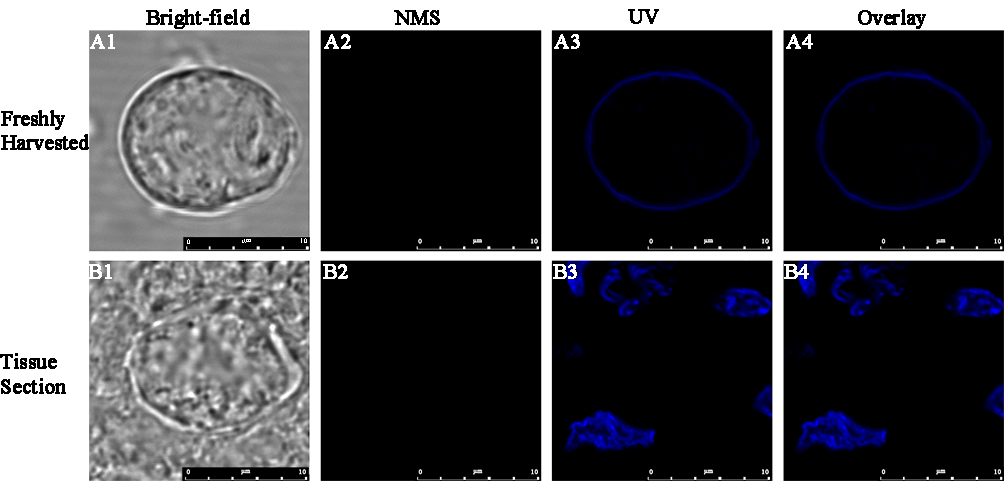

Supplement: Supplementary file 19 — Additional file 19: Figure S4. Negative control for immunofluorescence analysis of unsporulated oocysts freshly harvested (A) and in tissue section (B) of E. necatrix. NMS: normal mouse serum. [file 13071_2023_6076_MOESM19_ESM.tif]
